# Graphene-Based Polymeric Microneedles for Biomedical Applications: A Comprehensive Review

**DOI:** 10.1021/acsabm.4c01884

**Published:** 2025-02-10

**Authors:** Somayeh Moradi, Faezeh Nargesi Azam, Hossein Abdollahi, Nariman Rajabifar, Amir Rostami, Pablo Guzman, Payam Zarrintaj, Seyed Mohammad Davachi

**Affiliations:** † Department of Polymer Engineering, Faculty of Engineering, Urmia University, Urmia 57561-51818, Iran; ‡ Polymer Engineering Department, Faculty of Chemical Engineering, Tarbiat Modares University, Tehran 14115-114, Iran; § Department of Polymer Engineering and Color Technology, Amirkabir University of Technology, Tehran 15875-4413, Iran; ∥ Department of Chemical Engineering, Faculty of Petroleum, Gas and Petrochemical Engineering, Persian Gulf University, Bushehr 75169-13817, Iran; ⊥ Department of Biology and Chemistry, Texas A&M International University, Laredo, Texas 78041, United States

**Keywords:** Graphene, microneedles, biomedical application, transdermal, drug delivery

## Abstract

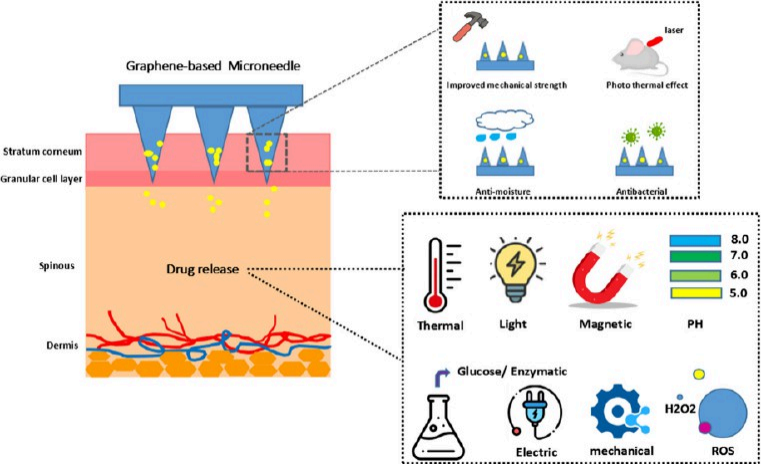

Transdermal drug delivery presents a promising noninvasive approach, bypassing first-pass metabolism and gastrointestinal degradation. However, the stratum corneum (SC) barrier limits drug absorption, necessitating the development of effective delivery systems. Microneedles, particularly polymer-based ones, offer a solution by penetrating the SC while avoiding critical nerves and capillaries. These microneedles are biodegradable, nontoxic, and easily manufacturable, making them a highly attractive platform for transdermal drug delivery. However, their clinical application remains limited due to suboptimal therapeutic efficacy and slow drug release rates. Recent advancements have introduced the incorporation of nanodrugs, such as nanoparticles and encapsulated drugs, into microneedles to enhance drug delivery efficiency. Among the materials explored, graphene and its derivatives, including graphene oxide (GO) and reduced graphene oxide (rGO), have garnered significant attention. Their exceptional mechanical strength, electrical conductivity, and antibacterial properties not only improve the mechanical performance of microneedles but also enhance drug release rates and biocompatibility. This review synthesizes the current state of microneedle technologies, focusing on the materials, fabrication techniques, and performance challenges. It particularly examines the potential of graphene-based microneedles, comparing them to traditional polymer-based microneedles in terms of drug release efficiency and stability. The review highlights key challenges, such as scalability, biocompatibility, and fabrication complexity, and suggests future research directions to address these issues. The incorporation of graphene quantum dots (GQDs) is identified as a promising avenue for improving drug release profiles, stability, and real-time tracking of drug diffusion. Finally, the review outlines emerging applications, including smart drug delivery systems, biosensing, and real-time monitoring, urging further exploration to unlock the full potential of graphene-enhanced microneedles in clinical settings.

## 1 Introduction

**Figure 1:**
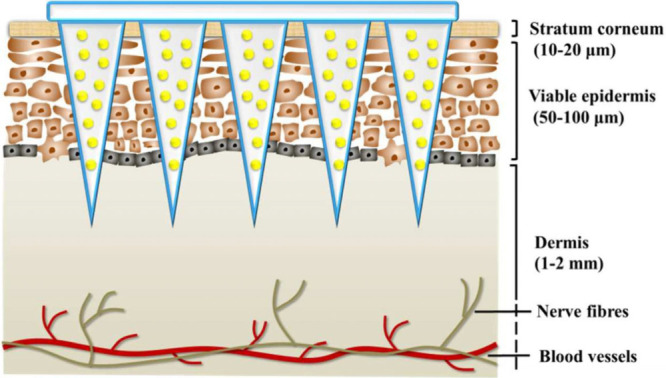
Illustration of transdermal drug delivery using a microneedle array containing medication. The array penetrates the stratum corneum (SC), enabling the drugs to reach the epidermal and dermal layers of the skin. Reproduced with permission from ref (1). Copyright 2017, Royal Society of Chemistry.

Oral delivery of medication is a convenient method, although it may not always be effective owing to enzymatic degradation in the liver or poor absorption. Hypodermic needle injections have been used as an alternative yet they possess drawbacks such as pain, invasiveness, and the need for trained administrators. Transdermal drug delivery (TDD) is another option that bypasses liver metabolism and can be self-administered without pain or invasiveness. Microneedles as three-dimensional structures with microscale lengths (usually less than 1000 m) have shown promise in TDD. They pierce the stratum corneum (SC) and produce a transient microchannel, diffusing passively external molecules into the skin. Microneedles can be tailored to a specific gauge, allowing them to reach a targeted depth without irritating underlying nerves in the dermis or causing damage to nearby blood vessels (Figure 1).^1^ Microneedles have been successfully applied in various fields, including angiogenesis, diagnosing and curing diseases, vaccine administration, and cosmetics, providing efficient yet painless TDD.^2−5^ Microneedles were introduced in 1976 as a hybrid approach between patches and needles to overcome these limitations.^6^ They safely and effectively deliver drugs across the skin barrier by generating micropathways in the SC for targeted release, allowing self-administration, high drug bioavailability, and skin penetration without touching nerve endings or blood vessels.^7^ Microneedles are available in a variety of sizes and shapes, with the type and tip shape playing a crucial role in enhancing drug penetration. They vary in dimensions, such as lengths ranging from 150 to 1500 μm and widths from 50 to 250 μm, with a tip diameter of 125 μm and heights spanning from 50 to 900 cm^2^.^8^

The material composition of microneedles, such as silicon, stainless steel, polymer, metals, and glass, significantly influences their strength and ability to pierce the skin effectively (Table 1).

**Table 1:** Short list of Microneedle Categories and Applications^a^

**Materials**	**Advantages**	**Disadvantages**	**Application**
**Silicon**	Biocompatible, durable, robust fabrication methods	Fragile	Solid, coated, hollow microneedles
**Glass**	Nonreactive, nonopaque, and inexpensive	Cumbersome fabrication, brittle	Hollow microneedles
**Ceramic**	Natural porous	Time-consuming processing method, fragile	Dissolvable, hollow microneedles
**Metals**	Biocompatible, highly conductive, catalytic activity for some nanometals	Expensive for some metals, risk of allergic reaction	Solid, coated, hollow microneedles
**Polymers**	Biodegradable, simple processing	Poor mechanical strength	Solid, hollow, coated, dissolvable, swellable microneedles

a Reproduced with permission from ref (9). Copyright 2021, MDPI.

The selection and utilization of microneedles vary based on their anticipated properties, cost considerations, and intended application sites. Among these materials, graphene-based microneedles have emerged as a promising option due to their superior mechanical properties and potential for controlled drug release. Metal microneedles, typically constructed from stainless steel or titanium, are renowned for their superior mechanical strength, enabling them to effectively penetrate tough skin barriers without the risk of breaking. Their high-precision manufacturing allows for reusable designs, which can be cost-effective for various applications. Additionally, metal microneedles can be engineered for controlled drug delivery, facilitating precise dosing essential for therapeutic efficacy. However, potential drawbacks include the risk of skin irritation or allergic reactions in some patients, which may limit their widespread use. Furthermore, the fabrication process for metal microneedles can be complex and costly, posing challenges for large-scale production. In contrast, ceramic microneedles are characterized by their high biocompatibility, which minimizes the likelihood of adverse reactions during drug delivery. They also exhibit excellent chemical stability, making them suitable for delivering sensitive drugs that might degrade when in contact with other materials. Nevertheless, ceramic microneedles are prone to brittleness, raising concerns about breakage during insertion and the potential for leaving fragments within the skin. Additionally, their mechanical strength is generally lower than that of metal microneedles, which can restrict their applicability in certain contexts.^10,11^

Graphene-based microneedles, however, offer unique advantages over metal and ceramic options. Graphene significantly enhances the mechanical properties of microneedles, facilitating their penetration through the skin barrier. The addition of graphene oxide notably strengthens dissolvable polymeric microneedles, increasing their strength by 10 to 17 times compared to nonreinforced versions. Furthermore, graphene-based microneedles enable controlled drug release through mechanisms like near-infrared (NIR) light activation, which is particularly advantageous for applications necessitating precise timing in drug delivery, such as cancer therapies. However, challenges such as high production costs and scalability issues persist, limiting the widespread use of graphene-based microneedles.^12,13^ Also, microneedles possess cylindrical, conical, or pyramidal tips and may be either solid or hollow.^14,15^ This diverse range of materials and shapes allows for customization to meet specific requirements in TDD. Considering the background of the emergence of microneedles and their fundamental benefits, progress in this area has led to the creation of diverse microneedles types. Based on device-based characteristics, they are classified into five groups: solid microneedles, drug-coated microneedles, dissolvable microneedles, hollow microneedles, and hydrogel-based microneedles. Microneedles made of polymers can be further categorized, such as dissolvable microneedles and swellable microneedles. Table 2 shows the common example of each group.^16,17^

**Table 2:** Typical Polymers for Microneedles Fabrication^a^

**Microneedle Type**	**Polymers**		**Fabrication Techniques**
**Dissolvable Microneedles**	Polysaccharides	• Dextran	• Micro molding
		• Sodium chondroitin sulfate	• Drawing
		• Hydroxypropyl cellulose (HPC)	• Lithography
		• Carboxymethyl cellulose (CMC)	• Continuous liquid interface production (CLIP)
		• Hydroxypropyl methylcellulose (HPMC)	• 3D printing
		• Sodium alginate	• Injection molding
		• Hyaluronic acid (HA)	• Hot embossing
		• Amylopectin	• Photolithography
			• Etching
	Others	• Gelatin	
		• Poly-γ-glutamic acid	Cross-linking via:
		• Polyvinylpyrrolidone (PVP)	• Lyophilization
		• Poly(methylvinylether/maleic anhydride) (PMVE/MA)	• Heating
		• Polyvinylpyrrolidone-polyvinyl alcohol (PVP–PVA)	• UV exposure
		• Poly (vinylpyrrolidone-co-met hacrylic acid) (PVP-MAA)	
		• PVP-cyclodextrin (PVP-CD)	
**Swellable Microneedles**	• Poly(methylvinylether/maleic anhydride)-polyethylene glycol (PMVE/MA-PEG)		
	• Poly(2-hydroxyethyl methacrylate) (PHEMA)		Cross-linking via:
	• Poly(styrene)-block-poly (acrylic acid) (PS-b-PAA)		• Lyophilization
	• Gantrez		• Heating
	• Acrylate modified HA (m-HA)		• UV exposure
	• Polyvinyl alcohol (PVA)		
**Biodegradable Microneedles**	Synthetic	• Polylactic acid (PLA)	• 3D printing
		• Polyglycolic acid (PGA)	• Stereolithography (SLA)
		• Poly(lactic-co-glycolic acid) (PLGA)	• Micro molding
		• Polycarbonate	• Photolithography
		• Polystyrene (PS)	• Deep-reactive ion etching (DRIE)
		• Polycaprolactone (PCL)	
	Natural	• Chitosan	
		• Chitin	• Two-photon polymerization
		• Silk	

a Reproduced with permission from ref (17). Copyright 2021, MDPI. Reproduced with permission from ref (18). Copyright 2024, MDPI. Reproduced with permission from ref (19). Copyright 2021, MDPI. Reproduced with permission from ref (20). Copyright 2024, MDPI. Reproduced with permission from ref (21). Copyright 2024, Taylor & Francis.

**Figure 2:**
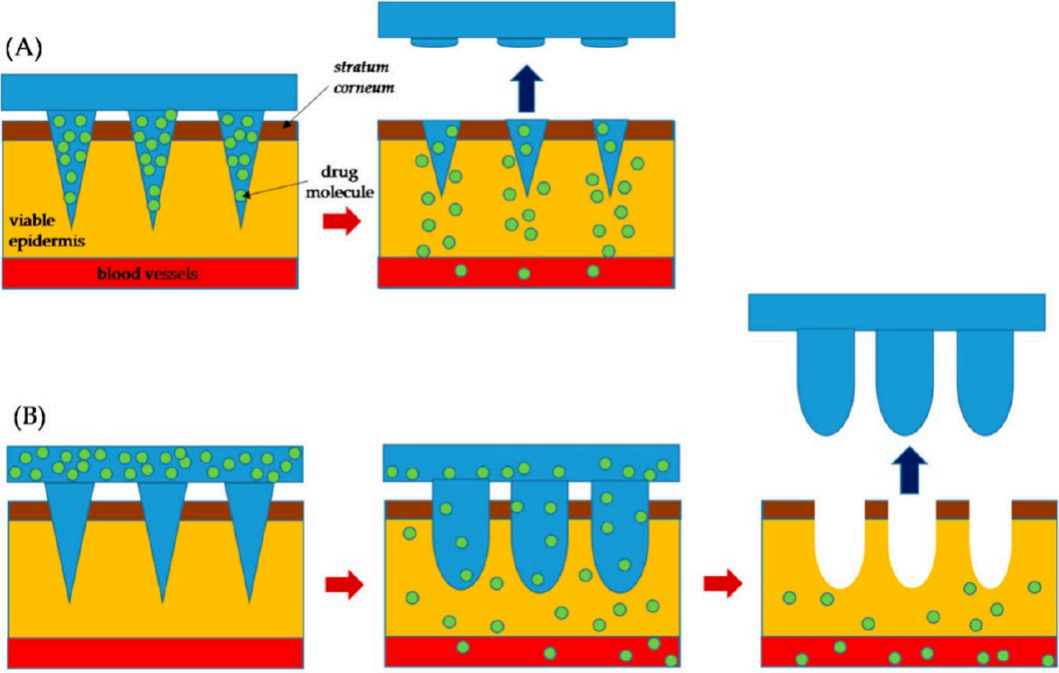
Illustration of drug delivery mechanisms of microneedles (A) Dissolvable microneedles. (B) Swellable microneedles. Reproduced with permission from ref (17). Copyright 2021, MDPI.

Dissolvable microneedles present a viable approach for drug delivery as they can be dissolved in the skin without generating biohazardous waste. The effectiveness of these microneedles relies on the properties of the polymer. Commonly used water-soluble polysaccharides like sodium chondroitin sulfate, hydroxypropyl cellulose (HPC), dextran, hydroxypropyl methylcellulose (HPMC), carboxymethyl cellulose (CMC), hyaluronic acid (HA), sodium alginate, and amylopectin are often utilized in the manufacturing of dissolvable microneedles. These polysaccharides contain hydrophilic groups or are branched, facilitating their dissolution in water.^1^ In contrast, swellable microneedles are made from cross-linked hydrogels that absorb fluid and expand when they contact with the skin, allowing them to draw out interstitial fluid and deliver preloaded medications. The drug delivery mechanism of dissolvable and swellable microneedles can be seen in Figure 2.

**Figure 3:**
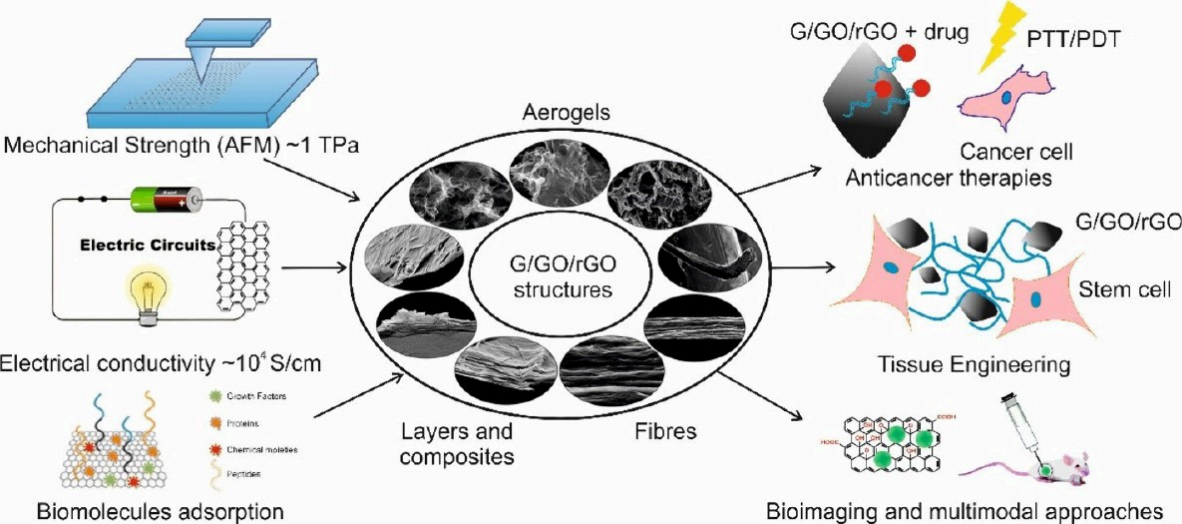
Schematic of graphene (G), graphene oxide (GO), and reduced graphene oxide (rGO) structures and their typical use. Reproduced with permission from ref (31). Copyright 2018, MDPI.

Coated microneedles, conversely, have drugs coated on their surface. When these microneedles penetrate the skin, the drugs dissolve into the interstitial fluid, facilitating drug delivery. Coated microneedles offer the versatility of delivering different types of drugs, including small molecules, DNA, viruses, proteins, and particles. However, their low drug loading capacity is a limitation that researchers attempt to overcome by using a layer-by-layer coating method.^4,22^ The matrix polymers used in dissolvable microneedles generally exhibit good biocompatibility. These polymers are not soluble or swollen in water, but they can be broken down by enzymes in the body. This results in a relatively slower drug release rate as the drugs are released after absorbing interstitial fluid. The degradation products of these polymers are harmless and removable from the body.^23^ Hollow microneedles serve as tiny conduits for drug delivery, allowing medication to flow through the channels and into the body as they pierce SC. However, the risk of blockage during skin penetration is a concern with hollow microneedles, as it can hinder drug delivery and increase the risk of infection. Various materials have been explored for fabricating hollow microneedles, including polymers, metals, silicon, and ceramics.^1,22,23^ Overall, dissolvable microneedles provide a versatile and efficient method for drug delivery. Various types of microneedles are being developed to address specific challenges and optimize drug release and penetration into the skin. Swellable microneedles developed in the skin are using cross-linked hydrogels, which extract fluid and releasing preloaded drugs. This is different from dissolvable microneedles, that undergo dissolution rapidly.^24,25^ Cao et al. have proposed a hybrid matrix based on poly(vinyl alcohol) (PVA) and maltose, which enhances mechanical strength and provides swelling to create micropores for drug release. The phase transition of PVA extends the drug release in vivo by an additional 8 h. However, the high-temperature fabrication method is a constraint, prompting the exploration of alternative materials like HA.^26^ Microneedles can be created using a variety of fabrication techniques, such as laser cutting, laser ablation, photolithography, dry etching, wet etching, three-dimensional (3D) printing, micro stereolithography, continuous liquid phase production, and two-photon polymerization. The choice of the fabrication method is recognized by factors such as the type of drug, dosage, desired pharmacokinetics and pharmacodynamics, design or materials of microneedles, and intended application.^24,25^ Graphene has gained substantial attention for its exceptional properties. It serves as a fundamental building block for carbon-based materials and possesses distinct characteristics. First, its charge carriers, known as Dirac Fermions, exhibit minimal scattering even under ambient conditions, allowing for efficient movement. Second, graphene is a zero-bandgap semiconductor in its two-dimensional form, impacting its electronic behavior. Third, it demonstrates a strong ambipolar electric field effect, enabling control over charge carrier concentration. Graphene also displays unique optical properties and high transparency to visible light. Its electronic, optical, and mechanical characteristics make it highly promising for various applications including electronics, sensors, nanoelectronic chips, and composite fields.^27^ Graphene has also found applications in medicine and biotechnology owing to its unprecedented potential, namely cell organization and growth, biological sensors, water purification, and nanomedicine. In addition, graphene derivatives have expanded applications.^28,29^ Examples of graphene-based materials include graphene oxide (GO),^30^ and reduced graphene oxide (rGO), with their applications and properties are shown in Figure 3.

**Figure 4:**
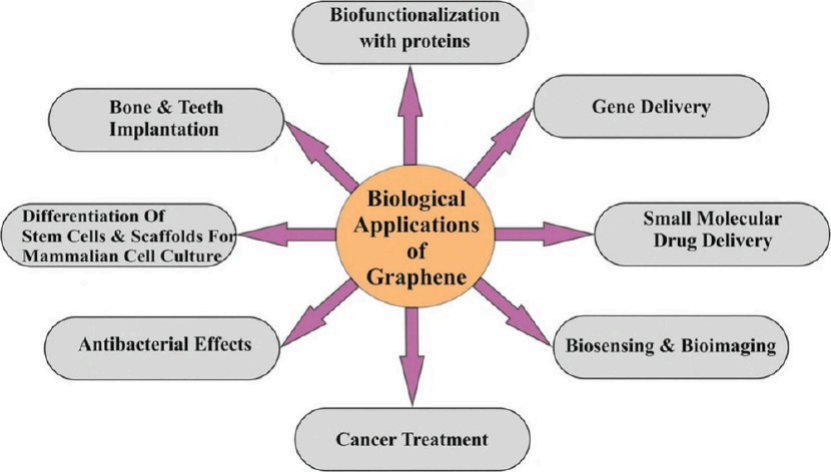
Graphene derivatives have expanded biomedical applications. Reproduced with permission from ref (32). Copyright 2018, Springer Nature.

Graphene is a functional material in composites, enhancing their characteristics and performance. It boasts excellent elasticity, strength, and porosity, positively impacting composites when integrated. The graphene-based materials can be actively used in biomedical applications, as shown in Figure 4. Certain types of graphene demonstrate increased strength under compression, especially under cross-linked conditions. The spongy structure of these graphene variants makes them perfect for reinforcing polymer matrices. This suggests that graphene matrices offer high strength, increased hydrophobicity, and superior conductivity.

For instance, Pang et al.^33^ developed a unique nanocomposite of CoFe_2_O_4_ nanoparticles with graphene nanosheets (CoFe_2_O_4_/GN), which they used as a counter electrode in dye-sensitized solar cells (DSSCs). A study carried out by Najafi et al.^34^ demonstrated a printable conductive ink for functional coatings in consumer electronics and biomedical applications, consisting of an oil-in-water emulsion of poly lactic acid (PLA) and graphene. The ink was formulated using different ratios of PLA as a binder and applied to fabrics using a rod-coating technique to reduce waste. Seung-Woo Kim and colleagues fabricated flexible, conducting polyurethane-silver/graphene composite fibers through a wet-spinning method. These fibers have potential applications in smart textiles, robotics, wearables, health monitoring, and biological systems for rehabilitation and performance enhancement.^35^ Jankovic et al.^36^ created novel Ag/HAP/Gr biocomposite coatings on titanium via electrodeposition, aiming to produce antimicrobial coatings for hard tissue implant applications. They intended to evaluate the potential of graphene-based microneedles as novel and efficient delivery systems for biomedical applications. This research centered on characterizing the physicochemical properties of graphene-based microneedles, exploring their capability for controlled drug delivery and biosensing. Furthermore, the study seeks to analyze the potential for graphene-based microneedles to enhance transdermal drug delivery, biosensing accuracy, and other biomedical applications, ultimately contributing to the development of advanced, next-generation biomedical technologies. Graphene materials exhibit significant advantages for microneedle applications, particularly in drug delivery systems. Their unique properties enhance drug loading capacity, control release rates, and minimize toxicity, making them superior candidates for microneedle fabrication. The following sections elaborate on these critical factors. Graphene-based nanomaterials possess a high surface area, allowing for substantial drug loading. For instance, GO can encapsulate a variety of therapeutic molecules effectively. Graphene materials enable controlled drug release through stimuli-responsive mechanisms, such as pH or temperature changes, which can be tailored for specific therapeutic needs. The integration of biocompatible polymers with graphene can further reduce potential adverse effects, ensuring safer applications in biomedical fields.^37^ Therefore, in this review, we focus on the literature related to this topic. First, we briefly introduce the concept of microneedles. We then discuss studies on graphene and its properties. We ultimately present research on the applications and limitations of graphene-based microneedles as novel and efficient delivery systems for biomedical applications.

## 2 Graphene

### 2.1 Overview of Structure and Applications of Graphene

**Figure 5:**
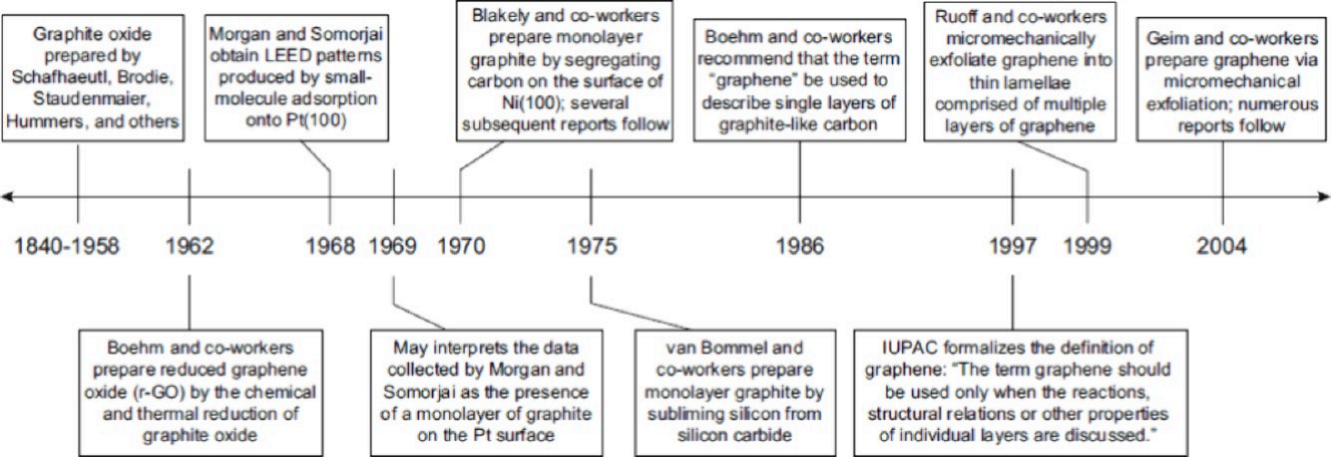
Selected events in the history of graphene. Reproduced with permission from ref (41). Copyright 2018, Springer Nature.

Carbon-based materials are renowned for their superior environmental and biological compatibility compared to inorganic materials, owing to the abundance of carbon, one of the most prevalent elements in our ecosystem. Specifically, graphite, a naturally occurring substance, has been utilized in our daily routines for centuries, demonstrating a consistent record of being nontoxic and devoid of significant toxicity concerns.^38^ Carbon exists in various forms, including diamond and graphite, which are three-dimensional allotropes with a history dating back to the 16th century. Fullerenes and carbon nanotubes, other forms of carbon, were discovered in the 1980s and 1990s. The existence of two-dimensional (2D) carbon allotropes was a subject of debate until 2004 (Figure 5) when Geim et al.^39,40^ successfully synthesized monolayer graphene. The 2010 Nobel Prize in Physics recognized their innovative work, establishing graphene as a highly sought-after material for research worldwide.^15^

**Figure 6:**
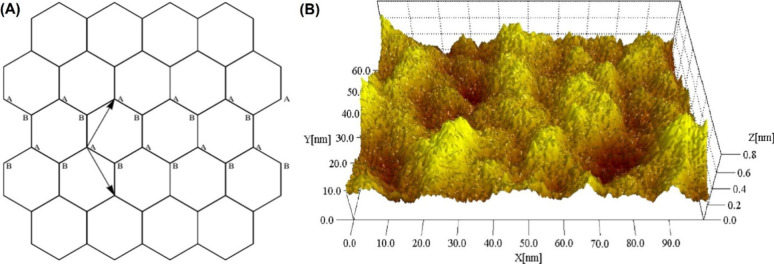
Graphene structure. (A) The hexagonal lattice of graphene is formed by overlaying two triangular sublattices, A and B. The basis vectors for this structure are defined as a_1_ = aand a_2_ = a, where a is the lattice constant, with a *= |*a_1_*| = |*a_2_*|*. The corresponding reciprocal lattice vectors are b_1_ = and b_2_ = Reproduced with permission from ref (45). Copyright 2010, IOPScience. (B) A stereographic representation of a large-scale STM image (100 × 62 nm^2^) of a single-layer graphene film deposited on a silicon dioxide substrate. The scanning tunneling microscopy (STM) conditions were set to a sample bias voltage of V_bias_ = 1 V and a tunneling current of I = 0.6 nA. To emphasize the surface characteristics, the vertical (Z) coordinate, with a scale of 0.8 nm, has been significantly magnified. Reproduced with permission from ref (46). Copyright 2007, National Academy of Sciences (PNAS).

Graphene is a 2D nanomaterial made from a single layer of carbon atoms in a hexagonal lattice, bonded through sp^2^ bonds.^42^ It is a million times smaller than the diameter of a human hair and only 0.334 nm thick, making it the thinnest material in the world.^43^ Graphene’s carbon atoms have sp^2^ hybridization, forming four bonds with a bond length of ∼0.142 nm. Graphene’s carbon atoms form 3 in-plane σ bonds and 1 out-of-plane π bond, creating a robust hexagonal lattice. The resulting hexagonal lattice forms a unique atomic-scale structure (Figure 6).^41,44^ Graphene’s unique properties stem from its sp^2^ carbon atom structure. Changes in this structure, like absent sp^2^ atoms or sp^3^-hybridized atoms, can alter its properties.

Graphene’s electrical conductivity is mainly due to the strong vertical bonds within its lattice structure. The stability of graphene is rooted in its tightly packed carbon atoms and unique sp^2^ hybridization, which merges p_*x*_, and p_*y*_ orbitals to form a robust π-bond. The remaining p_*z*_ electron enhances this bond, leading to the formation of π-bands and π*-bands. These bands are critical to graphene’s electronic properties, enabling the free movement of electrons within the half-filled band and contributing to its conductivity.^47^ Graphene is typically modified through chemical functionalization, enabling it to be dispersed in water and other solvents.

Table 3 lists different forms of graphene that result from functionalization or modifications, along with their applications. The surface atoms of graphene allow for easy attachment of biomaterials, but its tendency to cluster and cause oxidative damage poses a challenge. To overcome this, surface treatment and functionalization using techniques like deep eutectic solvents can be employed, enabling the attachment of specific functional groups. This functionalization unlocks new possibilities for drug delivery, leveraging graphene’s high surface-to-volume ratio to enhance its potential in this field.^48^

**Table 3:** Properties and Applications of Graphene^a^

**Property of graphene**	**Form of graphene**	**Applications**
**Mechanical properties and electrical conductivity**; high room-temperature mobility (∼200,000 cm^2^/V·s) and high electrical conductivity (ballistic electron transfer; high mobility), high strength (∼1100 GPa modulus, fracture strength ∼130 GPa), high surface-to-weight ratio (specific surface area)	Polyaniline-graphite electrode	Promotes damaged sites regenerate.
	Poly-3,4-ethylene dioxythiophene/graphene oxide (GO) composite film	It can be used an implantable device to modify the electrode site.
**Optical properties**: high light transparency	Combine ventral window with graphene sensor	The activity of the enteric nervous system is monitored and recorded by light stimulation.
**Thermal properties**: high thermal conductivity (thermal conductivity ∼3000 W/m·k out of plane)	Graphite lattice	Uses in drug delivery for neurological diseases.
	NGO-PEG	The tumors in the body are drastically removed by near-infrared laser irradiation
	Graphene	Photo thermotherapy isolates the amyloid B-protein fibers in Alzheimer’ s disease.
**Biocompatibility**: high sensitivity for chemical (physical properties can be chemically tuned)	Hydrogels containing graphene	Promotes neuron regeneration and support its differentiation.
	Graphene	Maintains the viability of neurons.
**Toxic properties**	Human umbilical cord Wharton’s jelly derived mesenchymal stem/stromal cells grown on reduced GO	Cells to show smaller and elliptical shape and differentiation into nerve cells.
	Reduced GO	The survival rates of A549 cells are reduced.
	GO	It leads to nervous system damage, increased

a Reproduced with permission from ref (48). Copyright 2023, Frontiers.

**Figure 7:**
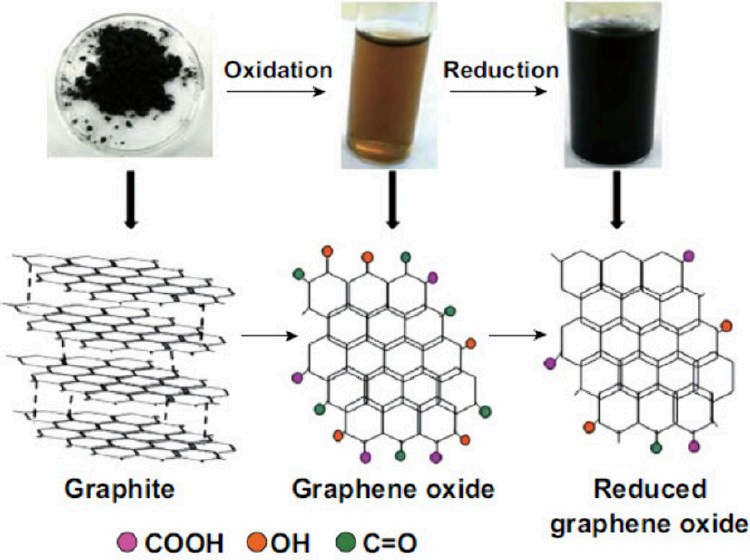
Process of graphene synthesis: conversion from graphite to rGO. Graphite is typically exfoliated and oxidized to form hydrophilic GO (brown solution). GO is further reduced to obtain rGO, which is less stable in water (black solution). Reproduced with permission from ref (50). Copyright 2019, Springer Nature.

GO is a single-layer material derived from graphite, usually produced through exfoliation. GO synthesis involves functionalizing sonicated graphite with acid and base. The surface of GO features functional moieties, including oxygen, epoxide, carbonyl, hydroxyl, and phenol. The primary distinction between graphene and GO lies in the attachment of oxygen atoms to carbon atoms in GO. rGO is produced by chemically or thermally reducing GO, resulting in a different material with unique properties.^49^ Process of graphene synthesis can be seen in Figure 7.

**Figure 8:**
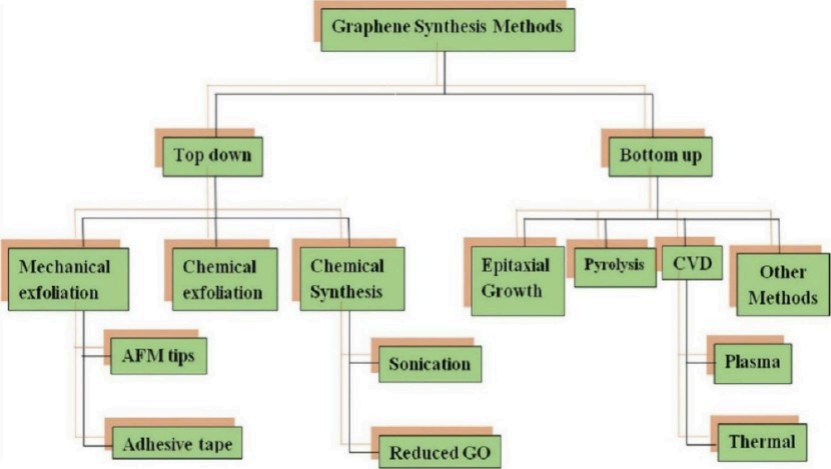
Methods of graphene synthesis. Reproduced with permission from ref (49). Copyright 2021, Elsevier.

Graphene is derived from GO upon eliminating the oxygen-containing functional groups through thermal or chemical reduction processes. Graphene synthesis can be achieved through two primary methods: top-down and bottom-up approaches, which more details about the synthesis is depicted in Figure 8. The top-down approach involves breaking down larger graphene sheets into smaller, more manageable pieces using electrochemical oxidation, chemical ablation, or plasma treatment. In contrast, the bottom-up method utilizes constructing larger graphene sheets from basic carbon precursors. In essence, the top-down approach starts with larger graphene structures and reduces them, while the bottom-up method builds up graphene sheets from smaller carbon source materials.^50^ Graphene, a semimetal, has an electronic structure similar to benzene rings, and its layered arrangement can influence its overall surface area. The structure is stable and can withstand external forces without reconfiguring atoms.^49,51^ These layers feature properties, such as high electron transport properties at ambient conditions, adjustable optical properties, quantum hall effect at room temperature, and tunable band gaps. In addition, graphene also has excellent properties, including high tensile strength (about 130 GPa) and flexibility (∼20%). It exhibits a low resistance value of 10^6^ cm (at 300 K) and also a significant charge carrier density (>10^12^ cm^2^). Depositing a single layer on a noncrystalline surface lowers the conductivity to 500–600 W/mK. It boasts a theoretically large specific surface area (2630 m^2^/g), high Young’s modulus (1.0 TPa), good optical transmittance (97.7%), excellent electrical conductivity, and can resist a current density of 10^8^ A/cm^2^.

Table 4 lists the main features of graphene and its nanostructures. Materials based on graphene show exceptional performance in electrochemical and optical applications, and can effectively attract and hold various aromatic biomolecules through strong interactions, making them well-suited for developing biosensors and drug delivery systems.^52^

**Table 4:** Physicochemical Properties of Graphene-Based Nanostructure^a^

**Property**	**Single-layer graphene**	**GO**	**rGO**
Young’s modulus	1000 GPa	220 GPa	N/A
**Fracture strength**	130 GPa	120 MPa	N/A
**Optical transmittance**	97.7%	N/A	60–90% depending on reduction agent and fabrication method
**Charge carrier concentration**	1.4 × 10^13^ cm^–2^	N/A	N/A
**Room temperature mobility**	∼200,000 cm^2^ V^–1^ s^–1^	N/A	N/A
**Thermal conductivity**	∼5000 W·mK^–1^	2000 W·mK^–1^ for pure 600 W·mK^–1^ on Si/SiO_2_ substrate	0.14–0.87 W·mK^–1^
**Electrical conductivity**	10^4^ S·cm^–1^	10^–1^ S·cm^–1^	200–35,000 S·cm^–1^

a Reproduced with permission from ref (41). Copyright 2018, Springer Nature.

### 2.2 Graphene in Biomedical Applications

**Figure 9:**
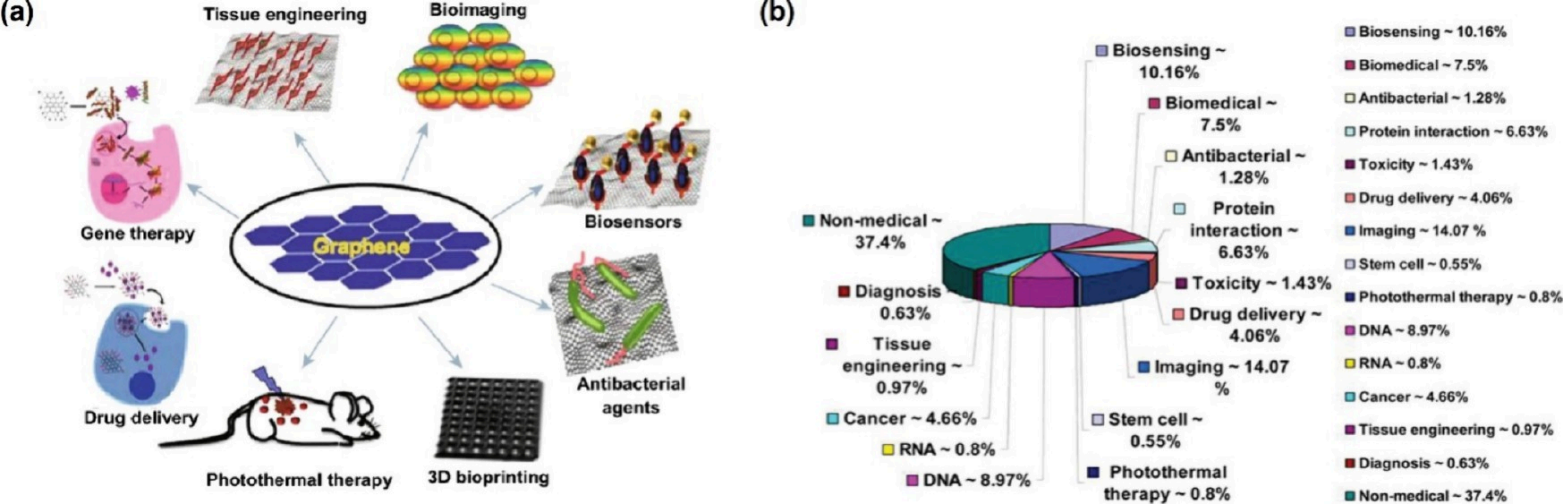
Graphene and graphene-based materials for biomedical applications. (a) Schematic of applications such as gene therapy, drug delivery, 3D bioprinting photothermal therapy, antibacterial agents, biosensors, bioimaging, and tissue engineering. Reproduced with permission from ref (50). Copyright 2019, Springer Nature. (b) Pie chart summarizing the proportional use of graphene, with key biomedical applications including biosensing, drug delivery, imaging, and DNA-related studies, alongside nonmedical uses. Reproduced with permission from ref (55). Copyright 2013, American Chemical Society.

The graphene family has sparked widespread fascination and interest among scientists across various disciplines, including composites and soft electronics. Graphene has achieved remarkable breakthroughs in biomedical, particularly in tissue engineering, targeted drug delivery,^53^ and biosensing, which is considerably propelling the progress of nanomedicine (Figure 9).^54^

Graphene is beneficial in diagnostics, where it acts as a nontoxic, biocompatible platform to anchor specialized recognition molecules, thereby amplifying the sensitivity and accuracy of diagnostic tools. Also, graphene’s outstanding electrical and optical properties enhance signal intensity, enabling more sensitive biomarker detection and imaging. The adaptability of graphene to be functionalized facilitates seamless integration with various nanomaterials, resulting in a hybrid system that leverages their combined strengths to achieve more accurate and advanced diagnostic outcomes. Gu et al. explored the application of graphene-based biosensors and bioimaging techniques for cancer diagnosis, focusing on the detection of specific cancer biomarkers. These biomarkers, including proteins, DNA, microRNA, and small molecules, can help identify cancer types and stages. For example, prostate-specific antigen (PSA) is usually used to diagnose prostate cancer, while breast cancer diagnosis often involves detecting specific genetic mutations like BRCA1 and BRCA2, as well as protein biomarkers such as CA 153, to determine the presence and progression of the disease. For liver cancer diagnosis, alpha-fetoprotein (AFP) is the primary biomarker used. Meanwhile, carcinoembryonic antigen (CEA), a protein heavily modified with sugar molecules, serves as a crucial biomarker for colorectal adenocarcinoma. Furthermore, certain metabolic byproducts, such as hydrogen peroxide (H_2_O_2_) and hydrogen ions (H^+^), are valuable indicators of cancer progression, providing additional insights into the disease’s development and advancement.^56^ Due to its properties, including high-energy transfer efficiency, extensive surface area, and compatibility with living systems, graphene is an ideal material for enhancing optical sensors. These advanced sensors can accurately detect a wide range of samples, from cells and proteins to small molecules, with heightened sensitivity and precision. Wang, the inventor of a groundbreaking sensor, announced a pioneering achievement in utilizing pure graphene to develop a highly sensitive sensor with diverse applications. He emphasized that leveraging graphene alone enables the creation of a cost-effective, flexible, and photosensitive sensor. Wang attributed the sensor’s success to its innovative trapped light nanostructures, which can retain light from electronic particles for extended periods, outperforming traditional sensors. This breakthrough capitalizes on graphene’s exceptional optical properties, providing researchers with invaluable insights and building on the material’s recent remarkable accomplishments.^57^ Graphene-based biosensors offer vast possibilities for detecting and monitoring various biological elements, including individual cells, cancer biomarkers, therapeutic drugs, proteins, and immune system interactions. These cutting-edge sensors can precisely detect subtle changes in the graphene surface structure and its interactions with biomolecules, enabling highly sensitive and accurate detection capabilities.^58^ Graphene and its derivatives stand out among various nanomaterials as an ideal alternative for creating scaffolds owing to the tunable properties they offer. Their nanoscale dimensions also mirror the size of cell surface receptors and extracellular matrix (ECM) features, making them well-suited for tissue engineering applications. Additionally, graphene-based materials demonstrate a remarkable ability to absorb serum proteins like fibronectin, lamina, and albumin, facilitating cell adhesion, growth, and differentiation. Notably, the structural characteristics and dimensions of graphene closely resemble key components of the extracellular environment, including ECM proteins (e.g., collagen), ion channels, signaling proteins, and cytoskeletal elements.^59^ As a result, adding graphene into polymer-based scaffolds enables the creation of customized biomaterials that mimic the properties of native tissues. Artificial scaffolds need to replicate the distinct mechanical characteristics and electrical properties found in different types of tissue. For instance, scaffolds used in bone regeneration require high stiffness (E > 109 Pa), whereas those for nervous tissue need to be much softer (E < 4102 Pa), and muscle tissue necessitates substrates with moderate stiffness (E > 104 Pa). By tailoring the properties of scaffolds to match those of the target tissue, researchers can create more effective and biomimetic supports for tissue engineering applications.^60^ Graphene, renowned for its exceptional strength and toughness, significantly enhances resilience, and tensile strength of polymeric scaffolds when integrated into them. By adjusting the graphene content, researchers can tailor the scaffold’s mechanical properties to closely match those of the extracellular matrix (ECM) of the target tissue. Moreover, graphene-based nanocomposite scaffolds exhibit nanoscale surface roughness, which facilitates cell attachment and influences cell shape, further promoting a biomimetic environment for tissue engineering applications.^59^ The ability of graphene to form a strong connection with growing neuronal cells is vital for their differentiation, as it guides the extension of developing neurites. Moreover, research has shown that tailoring the electrical conductivity of scaffolds is crucial for creating functional electroactive tissues. Graphene’s stable electrical conductivity in biological environments makes it an ideal addition to polymeric scaffolds, significantly reducing their electrical resistance. Scaffolds made with graphene can imitate and restore electrically active tissues such as those in the heart and nervous system. They can also improve the healing process for nonelectrically excitable cells that are exposed to electrical fields, like during bone repair and wound healing. Nevertheless, the exact impact of scaffold conductivity on cell differentiation remains unclear, as varying graphene content affects multiple scaffold properties, including roughness, cellular adhesion, and interactions with nutrients, growth factors, and waste, making it challenging to isolate the effect of a single scaffold feature on cell fate.^61^ The rising incidence of bone defects, caused by various factors, has made bone transplantation the second most common tissue transplantation. Certain nanomaterials, with structures similar to natural bone, offer unique properties such as enhanced mechanical strength, electrochemical capabilities, increased surface area, and suitable wettability. These properties provide structural support and regulate cell behavior, improving bone repair effectiveness. Graphene has shown great potential in bone repair owing to its electrical conductivity, large surface area, and atomic structure stability. Graphene can reinforce bone repair scaffolds, deliver electrical stimulation to promote bone formation and facilitate the adsorption of active substances. The hexagonal structure of graphene ensures stability during scaffold preparation and implantation. Numerous studies have demonstrated that graphene-containing composites can promote bone regeneration by regulating the extracellular microenvironment, enhancing osteoblast adhesion and mineralization, and inducing stem cell differentiation into osteogenic cells.^62^ In tissue engineering, numerous examples demonstrate the potential of graphene-based applications. For example, hybrid films composed of graphene and chitosan can restore tissue and enhance its functionality. Additionally, graphene modified with heparin chains has been found to retain its anticoagulant properties and exhibit a substantial increase in antifactor Xa activity, with a notable enhancement to 29.6 IU/mL compared to pristine GO at 1.03 IU/mL.^63^

## 3 Microneedle Technology Compared to Conventional Drug Delivery Methods

The epidermis (including the SC), dermis, and hypodermis create three main layers of skin. The SC is the most important barrier to transdermal drug delivery. To overcome this, various technologies have been developed to temporarily disrupt the SC and enhance skin permeability, including iontophoresis, sonophoresis, magnetophoresis, electroporation, and laser-microporation. However, traditional drug delivery methods often face challenges such as drug degradation, toxicity, and poor bioavailability. Microneedles have emerged as a promising solution, offering a painless, minimally invasive, and self-administered transdermal drug delivery system with improved bioavailability.^64^ The development of transdermal drug delivery systems has progressed through three generations.

The first generation focused on delivering drugs that could easily cross the skin without enhancement, leading to the creation of transdermal patches. These patches typically consist of multiple layers, including a drug reservoir, a semipermeable membrane, and an adhesive layer. The drug can be stored in a liquid or gel-based reservoir or added to a solid polymer matrix. However, first-generation transdermal delivery is constrained by the SC, which it poses a significant barrier to drug transport. Only drugs with specific properties, such as low molecular weight, lipophilicity, and effectiveness at low doses, are suitable for first-generation transdermal delivery. Despite these limitations, transdermal delivery offers advantages over oral delivery, including steady delivery profiles and improved bioavailability. Nevertheless, the strict requirements for drug candidates and the constraints of the lipid bilayers of the SC limit the use of first-generation transdermal patches.^64,65^

The next iteration aimed to improve the skin’s ability to absorb small molecules using driving forces, including iontophoresis, chemical enhancers, and noncavitational ultrasound. The goal was to temporarily disrupt the SC’s structure to increase permeability while avoiding damage to deeper tissues. Although advancements were made in developing clinical systems for both local and systemic use, this generation faced challenges in balancing enhanced delivery across the SC with the protection of deeper tissues. While successful for small molecules, the second generation had a limited impact on delivering larger molecules, highlighting the need for further innovation in transdermal delivery technology.^64,66^

The third generation of transdermal delivery systems focused on delivering macromolecules and vaccines across the SC using advanced techniques like thermal ablation, electroporation, cavitational ultrasound, microdermabrasion, and microneedles. Building on the second generation’s goal of enhancing skin permeability, the third generation introduced new methods to temporarily disrupt the SC without harming deeper tissues. These techniques improved the delivery of biotherapeutics and large molecules, increasing efficacy in clinical trials. However, some methods cause skin harm, discomfort, and side effects. Microneedles offer a solution, allowing for selective permeabilization of the stratum corneum without affecting deeper tissues. The third generation encompasses novel techniques that target the stratum corneum, enabling effective transdermal delivery of therapeutic proteins and vaccines in clinical trials.^66,67^

Microneedles are a technology that allows drugs to be delivered through the SC with minimal invasiveness, accessing the dermal microcirculation. They offer several benefits over traditional hypodermic needles, including reduced risk of infection transmission, decreased anxiety, and improved patient compliance. Microneedles can deliver a broad range of substances, from small molecules to large macromolecules, including oligonucleotides, vaccines, proteins, and hormones. Despite their advantages, transdermal delivery systems still face limitations in terms of available drug options. Table 5 summarizes the pros and cons of various transdermal drug delivery systems, highlighting their advantages and disadvantages.^9,68^

**Table 5:** Pros and Cons of Microneedle Transdermal Delivery^a^

	**Advantages**	**Disadvantages**
**Improved drug delivery**	(1) Drugs are delivered directly into the body through the stratum corneum	Drug dose is limited to the small size of the microneedle
	(2) Onset of drug action is rapid (since there are capillary bed and associated lymphatic vessels in the superficial dermis)	
	(3) Accurate drug dose is delivered by controlling microneedle formulations	
	(4) Microneedles avoid the first-pass metabolism	Sophisticated technologies are needed for manufacturing a microneedle patch with reproducibility
	(5) Microneedles enable high drug bioavailability	
	(6) It is effective foe vaccine delivery because of the abundance of immune cells in the dermis	
**Improved safety and patient compliance**	(1) Microneedles are painless and safe because are their small length and size	Temporary inflammation and allergy can be caused
	(2) The need for expertise is reduced for the patch application	
	(3) Microneedle patches reduce or eliminate biohazardous sharp waste	
**Improved manufacturing process and cost saving**	(1) The optimized solid-state formulation of the microneedle does not need the cold chain system	When the solid microneedles are applied, some part of the microneedles can be broken or left in the skin
	(2) Microneedle patches, which encompass the functionality of the drug, needle, and syringe, reduce the overall size of the drug package	Microneedle patches need a storage container for holding the microneedle patches hygienically without damage during distribution from the manufacturers to the patients
	(3) Microneedle patches save cost in terms of dose sparing, manufacturing, and logistics	

a Reproduced with permission from ref (68). Copyright 2021, Springer Nature.

**Figure 10:**
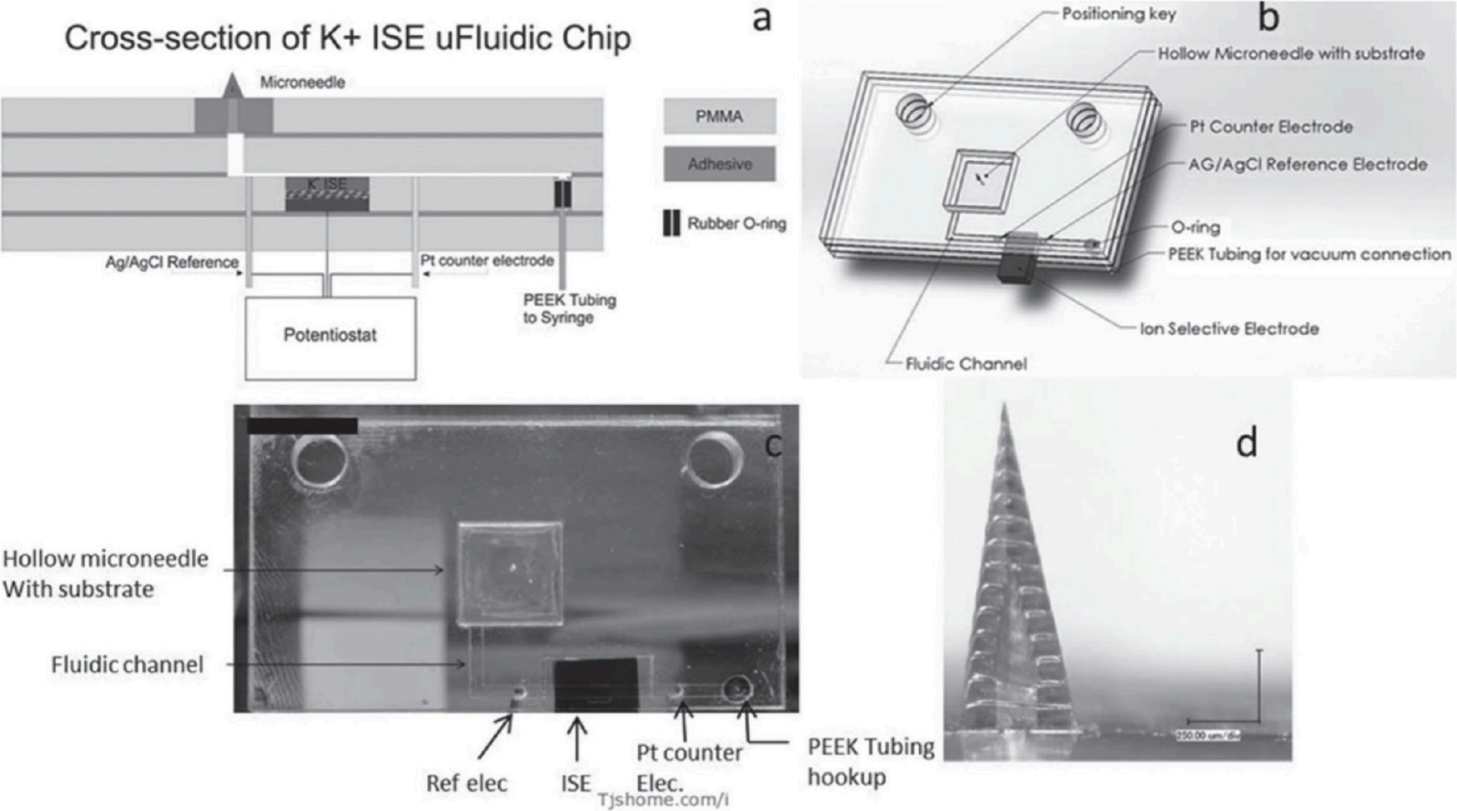
Design and fabrication of the K + ISE microfluidic chip. (a) Cross-sectional rendering created in CorelDraw. (b) SolidWorks illustration of the complete chip design. (c) Image of the fabricated microfluidic chip with integrated reference and counter electrodes (scale bar: 10 mm). (d) Optical image of a single hollow microneedle produced using two-photon lithography (scale bar: 250 μm). Reproduced with permission from ref (69). Copyright 2013, Wiley.

Measuring electrolytes is crucial for point-of-care diagnostics. Research by Miller presents a novel transdermal microneedle sensor for potassium detection, combining a hollow microneedle with a microfluidic chip to extract fluid and analyze it using a solid-state ion-selective electrode (ISE).^69^ The study compares porous carbon and graphene electrodes as transducers for ISEs, evaluating their electrochemical performance, stability, and selectivity. Porous carbon electrodes outperformed porous graphene electrodes, accurately measuring potassium levels in physiological concentrations while resisting interference from other ions. Notably, they showed no response to sodium ions. This pioneering platform, integrating microfluidics and microneedles, shows great potential for medical use, enabling the design of a body-worn sensor to continuously monitor vital signs including potassium levels (Figure 10).

## 4 Challenges in the Design and Fabrication of Microneedles

Microneedles with microscale dimensions (typically between 25 and 2000 μm in height) have emerged as minimally invasive tools for transdermal applications.^70−72^ Solid microneedles are simple to fabricate and have the potential for reuse. Coated microneedles feature a drug coating on their surface, facilitating controlled drug delivery at low doses. Hollow microneedles allow for the injection of larger drug quantities but may present challenges in terms of pressure resistance and tissue penetration. Dissolvable microneedles are cost-effective and readily dissolve under the skin, making them suitable for controlled drug delivery and vaccine administration. Various materials, including metals, silicon, ceramics, and polymers, can be used to fabricate microneedles. The choice of material is dependent on the desired properties and applications of the microneedles.^14,54,72^ Different fabrication techniques, such as laser cutting, etching, micromolding, and 3D printing, are employed based on the material and microneedle geometry. Among the 3D printing techniques, inkjet printing is commonly used for solid microneedles, allowing for precise drug loading and rapid release rates. Two-photon polymerization enables the fabrication of complex hollow microneedle structures with high resolution but currently has limitations in terms of speed and cost. Digital light processing (DLP) is a resin-based 3D printing method that offers fast print times and has shown promise in producing high-resolution hollow microneedles.^14,71−73^ The properties of microneedles differ depending on their type, requiring proper design based on factors such as drug dose, duration of release, delivery efficiency, packaging considerations, sharp waste management, and patch-wearing duration to ensure optimal performance and safety (Table 6).

**Table 6:** Suitable Microneedle Design^a^

**Parameters for microneedle type**	**Solid microneedle**	**Coated microneedle**	**Dissolvable microneedle**	**Hydrogel microneedle**
**Medicine dose**	High	Low	Low	High
**Onset of action**(pharma-cokienetic/pharma-namics)	Slow release by diffusion	Rapid dissolution	Depending on the formulation	Slow release by diffusion
**Delivery period**	Several hours	Several minutes	Several minutes to weeks	Several hours
**Delivery efficiency**	Some drug remains in the patch	High	High	Some drug remains in the patch
**Sharp waste generation**	High	High	No sharp waste	Swollen hydrogel Microneedle tip
**Packaging**	Separate packaging	High	High	High
Patch-wearing time	Several hours	Several minutes	Several minutes	Several hours

a Reproduced with permission from ref (68). Copyright 2021, Springer Nature.

## 5 Parameters Affecting Microneedle Insertion

**Figure 11:**
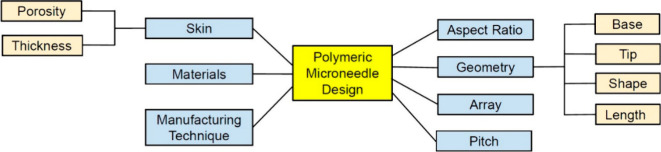
Effective parameters on polymeric microneedles. Reproduced with permission from ref (17). Copyright 2021, MDPI.

Effective skin penetration is a critical requirement for microneedle patches. To achieve this, it seems essential to consider the properties of skin, which can vary across different body parts and individuals. The success of microneedle insertion and penetration, designed to overcome skin elasticity, depends on several key factors including the microneedle’s geometry, base and tip diameters, length, and the spacing between microneedles (center-to-center distance). These parameters play a crucial role in ensuring optimal skin penetration as shown in Figure 11.^74,75^ In other words, a universal approach is not suitable for designing and developing microneedle applications, as the performance of microneedles in infiltrating the skin and delivering active ingredients varies. This encompasses various factors such as the shape and arrangement of individual microneedles, materials, method of administration, and the characteristics of the skin itself. Customizing the shape and composition of microneedles allows for precise control over their mechanical strength, penetration depth, and drug delivery profile, enabling optimization for specific medications and applications. This allows for tailored solutions to meet specific requirements.^76^

### 5.1 Geometry

**Figure 12:**
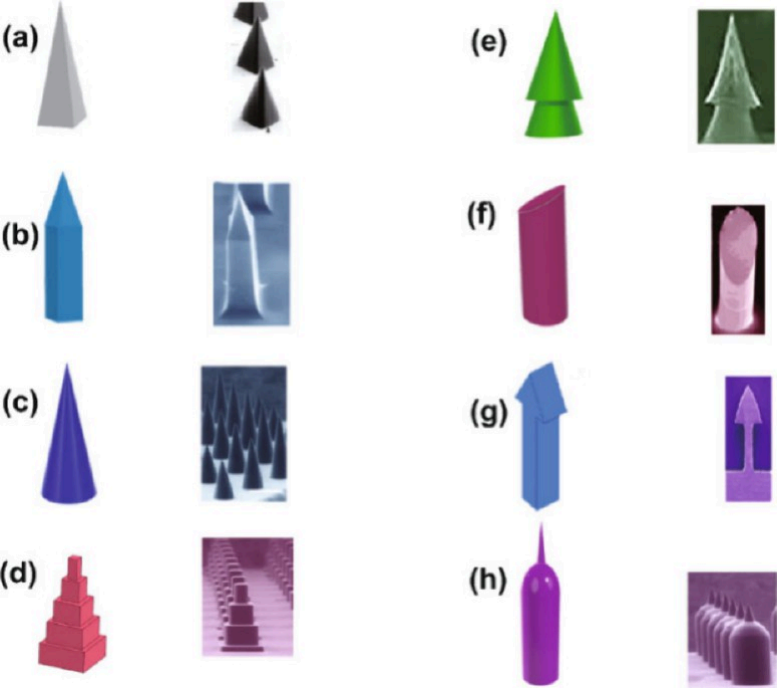
Microneedle designs and geometries, with schematics on the left and corresponding SEM images on the right, labeled (a) through (h), illustrating diversity in microneedle structures and construction. Reproduced with permission from ref (79). Copyright 2021, Springer Nature.

**Figure 13:**
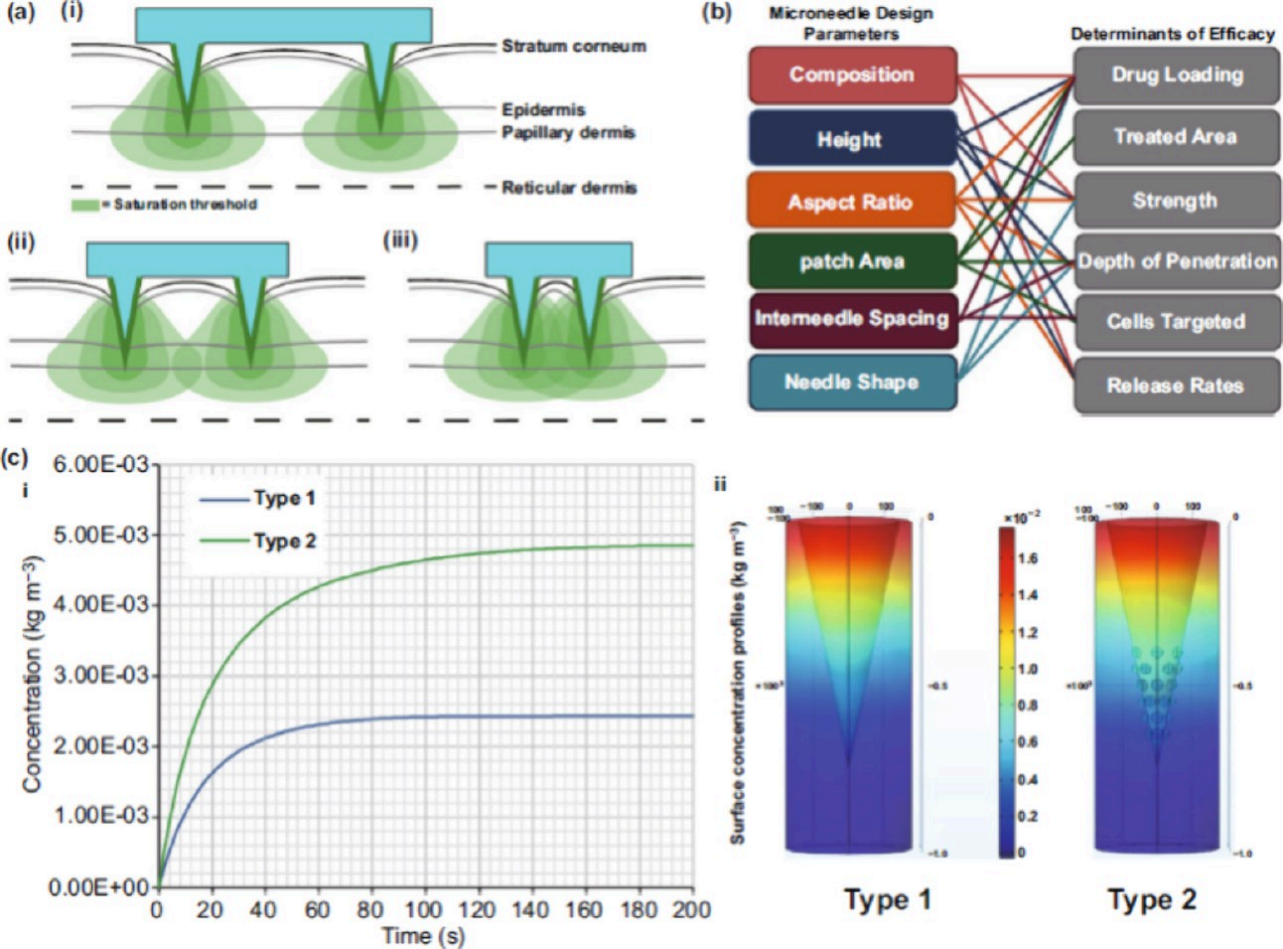
Optimizing microneedle design for enhanced dermal vaccine delivery and drug efficiency. (a) Variation of dermal vaccine delivery and cell activation with microneedle spacing and density as shown by the 2D schematic of large (i), optimal (ii), and small (iii) gaps between the microneedles. (b) drug efficiency and microneedle design parameters relation. (c) standard conical microneedle (type 1) compared to novel hemispherical design (type 2) for optimized drug delivery and surface concentration profiles. Reproduced with permission from ref (79). Copyright 2021, Springer Nature.

The shape of microneedles plays an integral role in clinical applications, affecting their mechanical strength and skin penetration ability. Research has shown a direct correlation between the number of vertices in the base of microneedle arrays (triangular, hexagonal, square) and their insertion depth, with triangular and square microneedles surpassing hexagonal ones (Figure 12).^77^ Cone-shaped microneedles have been proven optimal for delivering substances like ovalbumin, providing deeper insertion and faster dissolution for enhanced immune responses. To further improve drug delivery, a design featuring hemispherical convexities in the lower half of cone-shaped dissolvable microneedles has been suggested. The consideration of microneedle geometry is vital for effective clinical use.^78^ As the transdermal market expands, there is a growing need to clinically apply microneedles, driving the requirement for standardized tests to meet regulatory criteria. While ex vivo skin is often used to study drug delivery via microneedles, it is not reliable for assessing microneedle insertion consistency. Polymer film (PF) has emerged as a valuable tool for quality control of microneedle insertion. Computational modeling has been employed to recognize the most effective microneedle geometry for skin insertion and dermal vaccination. Research has shown that the ideal spacing between microneedles depends on the site, either epidermis or dermis, and the number of activated antigen-presenting cells, highlighting the importance of tailored design for effective application (Figure 13).^79^ Longer microneedles were found to be more effective in activating antigen-presenting cells in the dermis. A novel microneedle design, featuring a cone shape with hemispherical protrusions on the lower half, was proposed to mitigate the risk of inadequate drug delivery and enhance drug flux in cases of partial needle penetration. Furthermore, the force required for microneedle insertion was discovered to be significantly influenced by the angle of the tip and curvature radius, but only slightly affected by shaft width, with no impact from the radius. This insight can inform optimized microneedle design for improved performance.

### 5.2 Tip Diameter and Sharpness

Microneedle insertion is influenced by the tip diameter, with sharper tips facilitating smoother access to the skin. Blunt microneedles, measuring 60–160 μm, require more force (0.08–3.04 N) for controlled applications. Sharp-tipped microneedles enable precise insertion to the desired depth, a factor crucial for effective drug delivery. Smaller tip diameters (less than 15 μm) are more efficient for skin penetration, especially for vaccine delivery targeting specific cells in the skin layers. The sharpness of the tip affects the puncture force, with sharper tips requiring less force but also increasing the risk of breakage due to reduced structural strength. Therefore, balancing tip sharpness is essential for successful microneedle use in therapeutic applications.^80^

### 5.3 Length

The varying thickness of the skin’s layers across individuals can result in inconsistent particle insertion depths. The effectiveness of microneedle patches relies on how deeply they penetrate the tissue, as this impacts the skin’s ability to transport substances. For small drugs with high diffusion rates, creating surface pores through microneedle application may be sufficient for therapeutic effects. Nonetheless, for quick bloodstream delivery, deeper pores reaching the dermis, where capillaries are present, may be necessary. This could explain the range of microneedle lengths reported, including shorter microneedles and longer ones (up to 1000 μm) used to enhance insulin permeability in various studies.^81^ The production of microneedles depends on various parameters, the characteristics of materials, the shape and design of needles, the manufacturing process, and the intended use. A summary of the critical practical factors and considerations is tabulated in Table 7, which outlines the essential parameters to consider when fabricating microneedles.

**Table 7:** Effecting Parameters on the Fabrication of Microneedles^a^

**Parameters**	**Details**	**Practical considerations**
**Material properties**	Mechanical properties	Stiffness and brittleness affect material durability
	Chemical properties	Reactive materials need special handling
	Biocompatibility	Materials need to be safe for medical purposes.
**Device design**	Compatibility with drug formulations	Material should not affect drug potency
	Needle length and diameter	Microneedle size should be optimized for best results
	Needle shape and geometry	Microneedle shape impacts skin penetration
	Delivery target	Design considers site and action scope
**Quality control**	Inspection and testing	Tested to meet quality and design specs
	Precision and accuracy of alignment	Proper alignment ensures consistent drug delivery
**Cost**	Materials and manufacturing costs	Materials should be affordable and easy to process
**Regulatory requirements**	FDA regulatory	Microneedles require to meet regulatory safety standards

a Reproduced with permission from ref (83). Copyright 2023, MDPI.

### 5.4 Biocompatibility, Biodegradability, and Stability

The biocompatibility of microneedle systems is integral for their safe use in clinical settings. To guarantee human safety, microneedle products must undergo various tests assessing their compatibility with the body depending on how long they are used: up to 24 h, 24–30 h, and over 30 h. These tests encompass evaluations for cell toxicity, allergic reactions, skin irritation, and skin tissue reactions for brief exposure periods, and further assessments for genetic damage and prolonged systemic toxicity for extended exposure periods. Biodegradable materials in microneedles are preferred, as they can be safely broken down and removed from the body. Lately, researchers have concentrated on creating biodegradable polymer-based microneedle systems, which provide the benefit of encapsulating medication within the microneedle structure for gradual release into the skin via natural breakdown or dissolution in bodily fluids.^82^

### 5.5 Loading Capacity

The loading capacity of microneedle devices is limited, with coated microneedles only able to dispense a small bolus dose of approximately 1 mg of medication. Hollow microneedles offer continuous or on-demand dosing but may face obstruction issues due to compressed skin tissue. Despite microneedles’ potential to overcome skin barriers, their success relies heavily on passive diffusion, making it challenging to administer large doses and potentially resulting in significant dose loss on the surface of the skin. This has raised concerns about the suitability of the technology for certain clinical applications, such as vaccine distribution, where the precise dosage is crucial. Recent studies have shown that direct administration of vaccines to the epidermis and dermis can elicit immune responses with lower doses than traditional intramuscular injections.^84^ However, the effectiveness of this approach may be compromised if only a small amount of the delivered dose contacts the skin. Although not an unattainable challenge, achieving the required threshold dosage for immunity may be more difficult with microneedles, particularly for vaccines.^85^

### 5.6 Cost of Microneedle Fabrication

Microneedle technology needs improved manufacturing processes for large-scale therapeutic use. While exact economic impacts are unclear, clinical application may be expensive due to complex production and lengthy approval processes. Preclinical results are promising, but broader economic and health impacts remain uncertain. A study comparing microneedle and traditional subcutaneous measles vaccinations found microneedle administration could save $1 million per million children vaccinated. Microneedle patch cost-effectiveness depends on approval rates and performance versus traditional methods. Another study suggested these patches for flu vaccines would be cost-effective at $9.50 if administered by healthcare workers, with enhanced efficacy potentially making them cost-effective up to $30 in various scenarios. Given healthcare cost pressures, evaluating microneedle delivery system expenses during development is crucial. Success depends on fabrication techniques, materials, and scalability. Materials must be compatible with the payload to ensure optimal delivery without compromising therapeutic stability or bioactivity. Ideal production methods should allow easy, quick, and affordable adjustments to material and geometry parameters. Scaling up microneedle manufacturing requires strategic planning, including ensuring consistent production, mass production capability, and adaptability to various health concerns. Regulatory approval and clinical integration are also key factors for successful healthcare implementation of microneedle technology.^86^

## 6 Regulation of the Microneedle Patches

**Figure 14:**
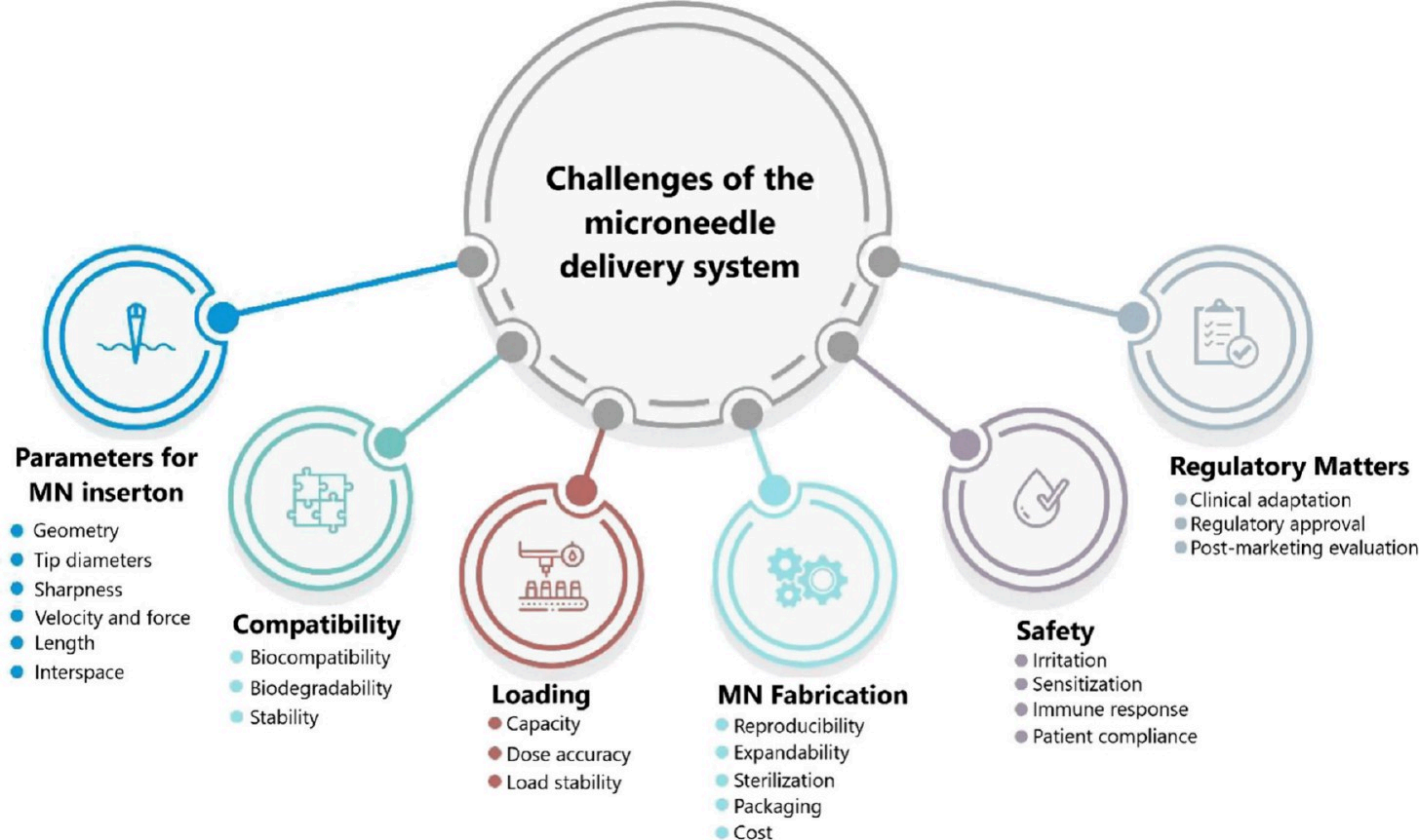
Factors influencing the development of microneedle-based delivery system. Reproduced with permission from ref (86). Copyright 2021, MDPI.

The FDA has raised concerns about the quality of submissions for microneedle combination products, particularly in stability testing and sterility validation. To secure approval, manufacturers must demonstrate clinical efficacy, device reliability, and repeatability through rigorous studies. Despite growing interest in microneedle-based products, the FDA submission process is complex due to detailed requirements for product analysis and testing. To ease commercialization, the FDA recommends integrating cGMP and quality control, clarifying licensing regulations, and streamlining regulatory processes by separating device development from drug formulation. Simplifying delivery via microneedle devices may alleviate regulatory challenges. Accurate classification of microneedle-based products is important for compliance, and adapting quality control methods may be necessary because of differences from traditional transdermal patches and hypodermic needles. Addressing regulatory requirements could soon make microneedle devices accessible, as seen with Zosano Pharma’s pioneering submission in 2020.^86^ As previously discussed in detail, there have been significant advancements in the construction and design of microneedles for targeted applications aimed at modifying various parameters to achieve specific objectives. In addressing certain challenges, polymeric microneedles have emerged as a promising avenue for further development. One of the most notable and impactful polymer families in this context is the graphene family, which will be elaborated upon in the subsequent discussion (Figure 14).

## 7 Fabrication Methods for Graphene-Based Microneedles

The fabrication of graphene-based microneedles involves various methods that cater to specific requirements such as precision, scalability, and material properties. While solvent casting is a commonly used technique due to its simplicity and cost-effectiveness, alternative methods provide distinct advantages for advanced applications. For instance, micromolding enables the precise formation of dissolvable microneedles, ensuring uniformity in size and shape.^87^ 3D printing has emerged as a versatile approach, allowing customization of microneedle designs for specific applications, including hollow or complex structures.^88^ Similarly, photolithography, often employed in the fabrication of silicon or graphene-based microneedles, offers high-resolution patterning suitable for intricate geometries.^89^

Other advanced techniques include etching to create high-precision features, hot embossing for scalable production of polymeric microneedles, and two-photon polymerization, which is particularly useful for fabricating detailed hollow structures.^90^ Laser cutting and laser ablation are also employed for their efficiency in shaping graphene-based microneedles with precision.^91^ The selection of a fabrication method depends on factors such as the required mechanical properties, drug loading capacity, and the intended application of the microneedle system. A combination of these techniques, tailored to the specific requirements of graphene-based microneedles, enables the development of optimized designs for biomedical applications.^68^

## 8 Graphene Properties for Microneedle Applications

The rapid growth in the adoption of graphene in biomedical engineering accounts for its exceptional characteristics, which include outstanding performance in biological compatibility, electrical conductivity, optical clarity, and thermal management. Collectively, these properties surpass those of commonly used biomaterials, thereby opening a broad spectrum of applications previously considered the realm of science fiction. The incorporation of nanoparticles with microneedles serves two primary functions, either to inhibit bacterial growth or to deliver therapeutic drugs. To achieve antibacterial properties, a range of antimicrobial nanoparticles are utilized, including metals like gold, silver, zinc oxide, and copper oxide, as well as carbon-based substances like graphene and nanotubes, and polymers such as quaternary ammonium, chitosan, and synthetic materials with cationic or water-repelling components.^19^ Graphene has established itself as a premier nanomaterial in biomedical engineering, seamlessly integrating biology, electronics, and nanoscience to pioneer innovative diagnostic and therapeutic approaches. The swift ascendance of graphene in biomedicine is rooted in its extraordinary attributes, which harmoniously blend exceptional electrical, thermal, optical, making it an ideal candidate for advancing medical technologies.^92^ The ease of synthesis and excellent dispersion process capability make graphene an inexpensive and appealing choice for large-scale applications in electrochemical analysis.^92,93^ Graphene consists of a solitary layer of carbon atoms arranged in a repeating hexagonal pattern, serving as a fundamental building block for a wide range of carbon-based materials. It can be transformed into different dimensional configurations, including 0D fullerenes, 1D carbon nanotubes, as well as 3D graphite crystals. The high surface area of graphene enables effective interactions with cells and offers numerous binding sites for biomolecules. Its distinctive electronic and optical properties, stemming from its unique band structure, combine the benefits of both metals and semiconductors. As a result, graphene’s bioactive, transparent, and zero-bandgap semiconductor nature enhances the performance of various biosensors, including electrochemical, field-effect transistors, and optical sensors, leading to improved detection capabilities for enzymes, DNA, and immune responses.^86,92,93^ Scientists explore the potential of graphene as a superior reinforcing material in biomedical applications, leveraging its exceptional strength, lightness, and unique 2D hexagonal structure, where each carbon forms strong bonds with its three adjacent atoms. Composites based on graphene can be integrated with various nanofabrication methods, allowing for customizable shapes and morphologies that mimic distinct cellular environments, including fibrous tissues and cellular structures, making them highly versatile for biomedical use. The development of microneedle technology for real-time transdermal analyte detection is still in its infancy. Nevertheless, biosensors utilizing nanomaterials have made significant strides in detecting biomolecules, capitalizing on the distinct properties of nanomaterials, which differ from their bulk counterparts. Nanomaterials come in various forms, including nanoparticles, nanowires, graphene, and aerogels, each with unique size-dependent characteristics. The incorporation of these nanoscale materials in sensor design holds immense promise for transforming the biosensing field, offering unprecedented opportunities for innovation and advancement. Current research is focused on integrating nanomaterials with microneedles to create transdermal electrodes. The application of graphene in transdermal biosensors presents a fascinating opportunity. Graphene, a 2D material with a unique sp^2^ hybridization, was first extracted by Novoselov et al.^39,94^ employing the scotch tape method, earning Geim and Novoselov the 2010 Nobel Prize in Physics. Graphene boasts an impressive combination of high surface area, mechanical strength, outstanding electrical conductivity, as well as rapid electron and carrier mobility. These attributes make graphene an optimal material for creating sensitive electrical surfaces and detecting target molecules. Furthermore, various graphene derivatives exist, including pristine graphene, GO, rGO, porous graphene, graphene quantum dots, and 3D graphene, offering a range of possibilities for biosensing applications.

## 9 Survey of the Recent Graphene-Based Microneedles for Biomedical Applications

**Figure 15:**
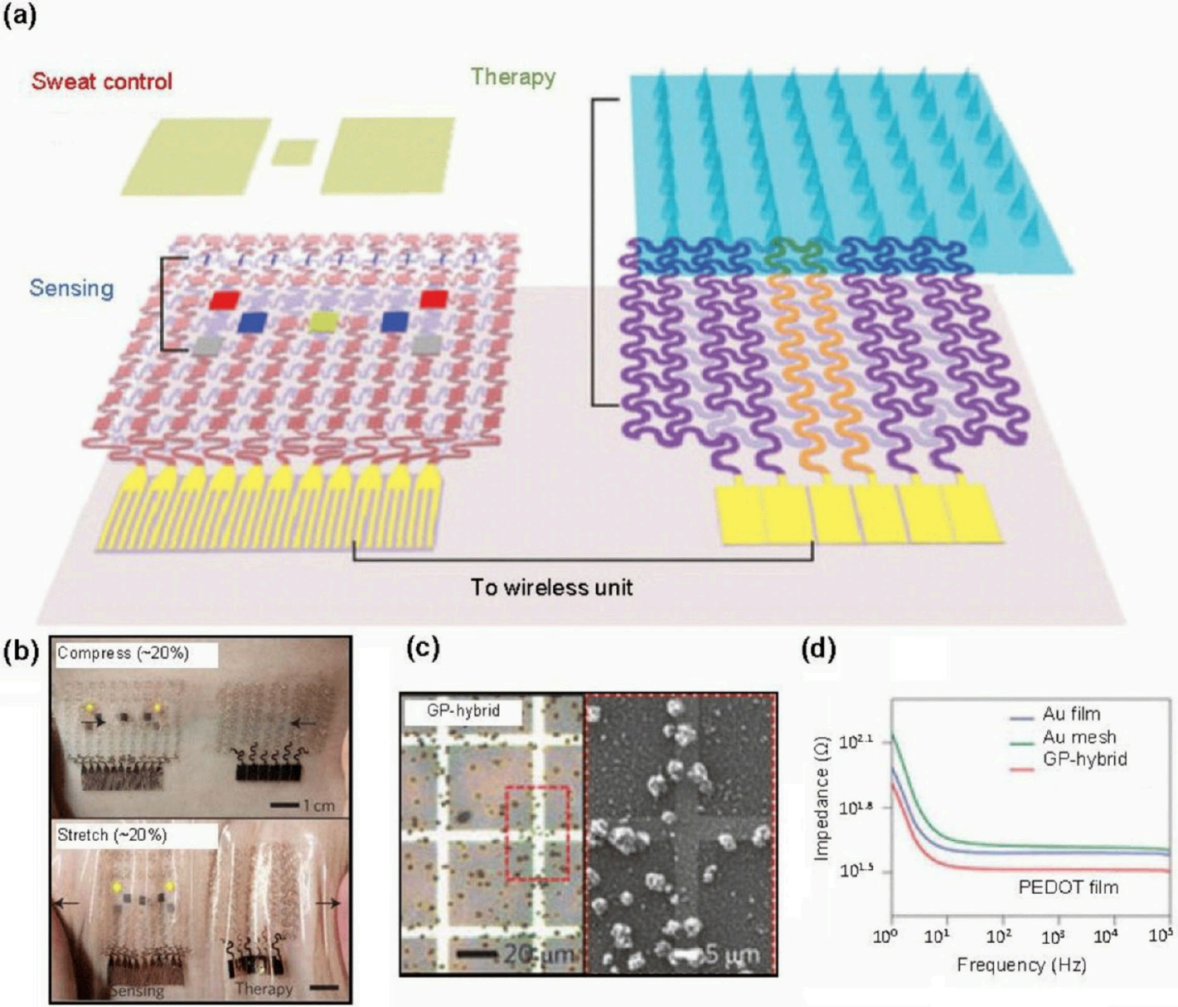
Diabetes monitoring system using graphene-based microneedles. (a) Schematic of a diabetes patch sensitive to sweat. (b) A wearable patch on human skin, under 20% compressive stress (top) and tensile strain (bottom). (c) Graphene-hybrid patch with PEDOT electrodeposition, visualized through optical microscopy (left) and SEM imaging (right). (d) Bode plots of the three electrodes in PBS after PEDOT electrodeposition. Reproduced with permission from ref (97). Copyright 2018, MDPI.

Over the past decade, scientists have made significant strides in exploring the biomedical applications of cutting-edge nanomaterials. Notably, graphene-family nanomaterials (GNFs) have emerged as a versatile tool in different biomedical domains, containing targeted drug delivery, biosensing, cancer therapy, tissue engineering, and more. As research on GNFs continues to advance, it is poised to revolutionize the field of biomedicine, opening up new avenues for innovation and transforming current practices.^95,96^ A flexible patch device, made up of a twisted dual layer of gold mesh and gold-doped graphene, allows for smooth and efficient electrochemical signal conduction.^97^ This device features an array of sensors, namely temperature, pH sensors, and humidity, as well as a heater and polymeric microneedles for transcutaneous drug administration. In experiments with diabetic mice, the patch effectively lowered blood glucose levels when activated by heat, using metformin as the antidiabetic medication. The utilization of soft materials enables seamless integration with human skin, enhancing sensor accuracy and drug delivery efficacy. By linking the device to a portable power source and data transmission module, diabetes management can be taken to the point of care, enabling convenient and accessible treatment. This technology shows promise for treating chronic diseases like diabetes mellitus. Lee et al.^98^ have proposed a groundbreaking, wearable device that combines the diagnosis and treatment of diabetes in a single platform. This innovative device integrates various sensors, such as pH and temperature, with thermoresponsive microneedles for drug delivery on a flexible substrate made of silicone. The device’s soft, serpentine design ensures comfortable, conformal contact with the skin, even under 20% tensile or compressive strains. The graphene-based hybrid material (GP-hybrids) enhances electrochemical properties, allowing for precise sensing and targeted drug delivery. When high glucose levels are detected, the heating element of the device dissolves the microneedles, releasing the drug into the bloodstream. In tests on diabetic rats, the therapeutic patch significantly lowered blood glucose levels compared to control groups, demonstrating its potential for effective diabetes management (Figure 15). Wearable biosensors provide numerous advantages, including compact size, robustness, cost-effectiveness, rapid response times, and minimal maintenance requirements. A key benefit of wearable sensing technologies is their capacity to deliver point-of-care diagnostics in remote and resource-constrained areas, such as rural regions and developing countries, where access to healthcare infrastructure and trained professionals for operating diagnostic equipment is scarce, enabling timely and accessible disease monitoring.

**Figure 16:**
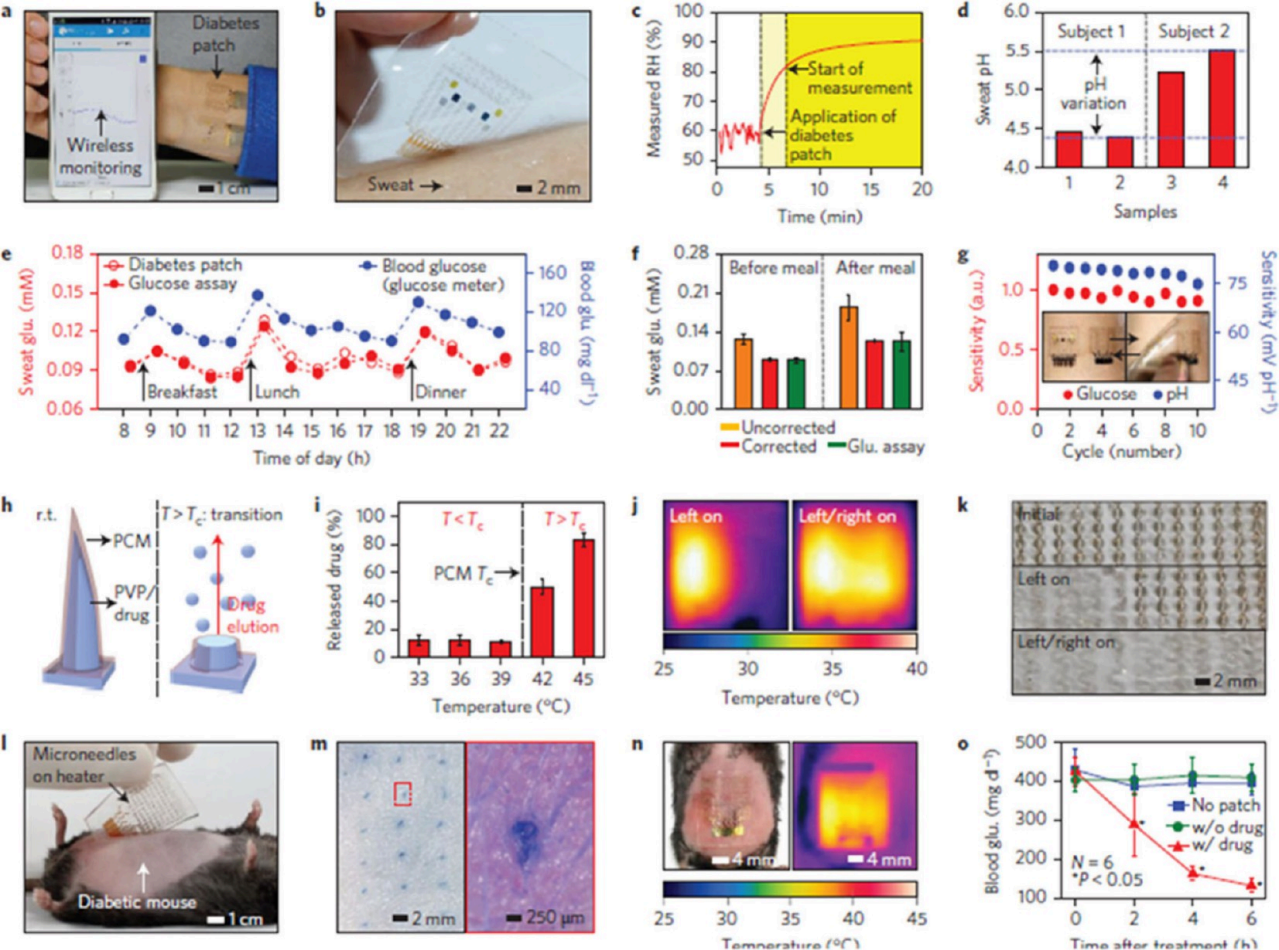
In vivo diabetes tracking patch for glucose level monitoring. (a) Image of the applied patch to a hand-held electrochemical testing device. (b) Image of a GP-hybrid wearable device applied on the skin. (c) Measured RH by the integrated patch. (d) Measured pH in two human sweat samples. (e) Tracking glucose levels in human sweat and blood over 24 h. (f) Comparing glucose levels with commercial test results, accounting for pH variations. (g) Consistent accuracy of glucose and pH sensors after several uses. (h) Schematic of bioresorbable microneedles. (i) Temperature-dependent release of drugs from microneedles. (j) Stepwise drug release captured by an infrared camera. (k) Image of the gradual dissolution of the microneedles. (l) Image of the applied heater with the microneedles. (m) Image of diabetic mouse skin stained with trypan blue (left) and its close-up view (right), showing tiny holes created by microneedle penetration. (n) Visible light image (left) and infrared thermal image (right) of the patch, showing heat activation. (o) Blood sugar levels in diabetic mice that received treatment. Reproduced with permission from ref (98). Copyright 2016, Springer Nature.

In recent years, wearable bioelectronics utilizing transdermal biosensors have gained attention. These devices make use of nanomaterials and have been developed in different forms. One notable example is the incorporation of microneedles into wearable biosensors, which has been widely explored.^73^ Graphene can be functionalized with biomolecules like enzymes and antibodies, expanding its potential for biomedical uses. Notably, researchers have developed wearable tactile sensors for human-like robots, featuring 3D microstructured graphene combined with polydimethylsiloxane (PDMS).^14^ These sensors demonstrate exceptional sensitivity, flexibility, and stability, enabling accurate detection of capacitive pressure with a low detection limit, paving the way for innovative applications in robotics and beyond. Wang et al.^99^ created a microneedles array sensor for real-time monitoring of abscisic acid (ABA) levels in plants. The sensor features a unique combination of Au@SnO_2_-vertical graphene (VG)/Ta microelectrodes, enabling precise quantification of ABA through direct electrocatalytic oxidation. Its compact design, wide pH tolerance, low detection limit, and broad linear concentration range make it an ideal tool for in situ ABA detection in plants. The effectiveness of the sensor was successfully demonstrated using cucumbers as test samples, showcasing its potential for accurate ABA monitoring in various plant species. The creation of point-of-care testing (POCT) for clinical biomarkers is vital for effective health monitoring and prompt treatment. Nevertheless, there is a need for biosensing assays that can detect biomarkers without relying on costly external equipment and reagents. Blood-based assays are particularly challenging due to the invasive blood collection process and the need for subsequent processing. In this regard, Keyvani et al. introduced an innovative solution: a versatile assay that employs hydrogel microneedles (Hmicroneedle) for minimally invasive interstitial fluid (ISF) extraction, combined with a graphene oxide-nucleic acid Hmicroneedle-GO. Nucleic acid (NA) based fluorescence biosensor for on-site biomarker detection, offering a promising alternative for convenient and efficient health monitoring. The Hmicroneedle-GO.NA assay is enhanced by a portable detector, facilitating a comprehensive point-of-care testing (POCT) process. Their system effectively measured four critical biomarker-glucose, uric acid (UA), insulin, and serotonin in laboratory tests, and successfully detected glucose and UA in real-time in vivo experiments.^100^ Building on their previous work, which introduced an optical Hmicroneedle biosensor for reagent-free analysis and on-microneedle detection without external processing, they have identified limitations in the displacement strand-based sensing approach, which restrict its adaptability and versatility. To overcome the existing limitations and streamline the fabrication process, they created an innovative Hmicroneedle assay that integrates GO.NA optical sensors for point-of-care testing (POCT) and sensing. This GO.NA optical sensor features GO nanosheets linked to (NA) with a fluorophore modification. When the target biomarker is not present, the NAs bind strongly to GO, causing the fluorophore tag to be quenched, resulting in a detectable change in the fluorescence signal. Conversely, when a specific biomarker is present, the nucleic acids (NAs) bind to their target, triggering a conformational change that moves the fluorophore tag away from the GO, resulting in the recovery of fluorescence and the generation of a detectable optical signal. The GO.NA assay showed accurate analytical detection in both laboratory and real-world settings, and its effectiveness was further evaluated in diabetic rat models for the in vivo detection of glucose and uric acid (UA), demonstrating its potential for precise biomarker monitoring. The integration of the GO.NA optical sensor with Hmicroneedle presents a promising approach for POCT and clinical biomarker detection (R3).^100^ The pandemic and potential future epidemics have created a pressing need for innovative vaccines, driving interest in subunit vaccines due to their affordability and safety profile. A novel vaccine adjuvant system was proposed, with combining CpG 1018 and GO to transport the Receptor-Binding Domain (RBD) of the SARS-CoV-2 spike protein. The resulting GO-based complex booster nano vaccine (GCR) showed enhanced antigen loading and encapsulation efficacy. Vaccination with GCR elicited a strong RBD-specific antibody response and a Type 1 Cellular response.^101^ Furthermore, a microneedle patch vaccine (MGCR) based on GCR was developed, demonstrating similar immune responses and sustained antibody levels over time. The MGCR microneedle patch vaccine also exhibited stability at room temperature for extended periods, enabling easy administration without medical supervision, thus improving vaccine distribution efficiency. Overall, this pioneering system presents an auspicious solution in order to enhance vaccine accessibility in resource-constrained regions and offers a potential vaccination strategy against SARS-CoV-2 and possible pandemics in the future.^101^ The GO-based microneedle patch vaccine system has demonstrated encouraging outcomes in inducing robust immune responses and stability, which could substantially enhance the accessibility rate of vaccines in low-paid areas, ultimately bridging the gap in global healthcare access. This approach holds potential for combatting SARS-CoV-2 and other future pandemics. The study also highlights the importance of pattern recognition receptors (PPR) agonists, such as TLR4 and TLR9, in vaccine adjuvant systems, further contributing to the development of effective vaccines against infectious diseases.^101^ While graphene has immense promise in microelectronics, its advantage in biosensing is restricted due to the scarcity of electrochemically active defects, especially when produced using the chemical vapor deposition method. However, research by Lee et al.^98^ reveals that gold-doped graphene, coupled with a gold mesh, significantly enhances electrochemical activity compared to pure graphene. This innovation paves the way for developing a wearable device that tracks diabetes through sweat analysis and provides personalized feedback therapy. The flexible device boasts a wavy two-layer structure that combines gold mesh and gold-doped graphene, enabling effective transmission of electrochemical signals. The patch includes sensors, such as pH and temperature, as well as thermally activated polymeric microneedles for transcutaneous drug delivery. Experimental results demonstrate the patch’s ability to thermally release Metformin, reducing the level of glucose in diabetic mice’s blood. This study unveils a next-generation class of diabetes monitoring and treatment solutions that harness the power of functionalized graphene, demonstrating advancements in device engineering, soft materials, and holistic system design (Figure 16). Biochemical sensors made with graphene and Ag/AgCl counter electrodes demonstrate improved electrochemical performance for detecting key biomarkers in human sweat. The combination of graphene-gold interconnections and physical sensors enables efficient signal transmission through the stretchable array. This integrated system monitors biomarkers, physiological cues, and sweat levels, while also enabling transdermal drug delivery, achieving a comprehensive closed-loop treatment for diabetes. The device detects humidity levels to activate glucose sensing, which is adjusted for accuracy by pH and temperature measurements. When glucose levels are high, embedded heaters dissolve phase change materials, releasing Metformin through bioresorbable microneedles for transdermal delivery. The use of flexible, conformal materials and wireless connectivity enhances the practicality of this patch system. These advancements in nanomaterials and devices pave the way for novel approaches to combating chronic diseases like diabetes, offering promising solutions for treatment.^98^

**Figure 17:**
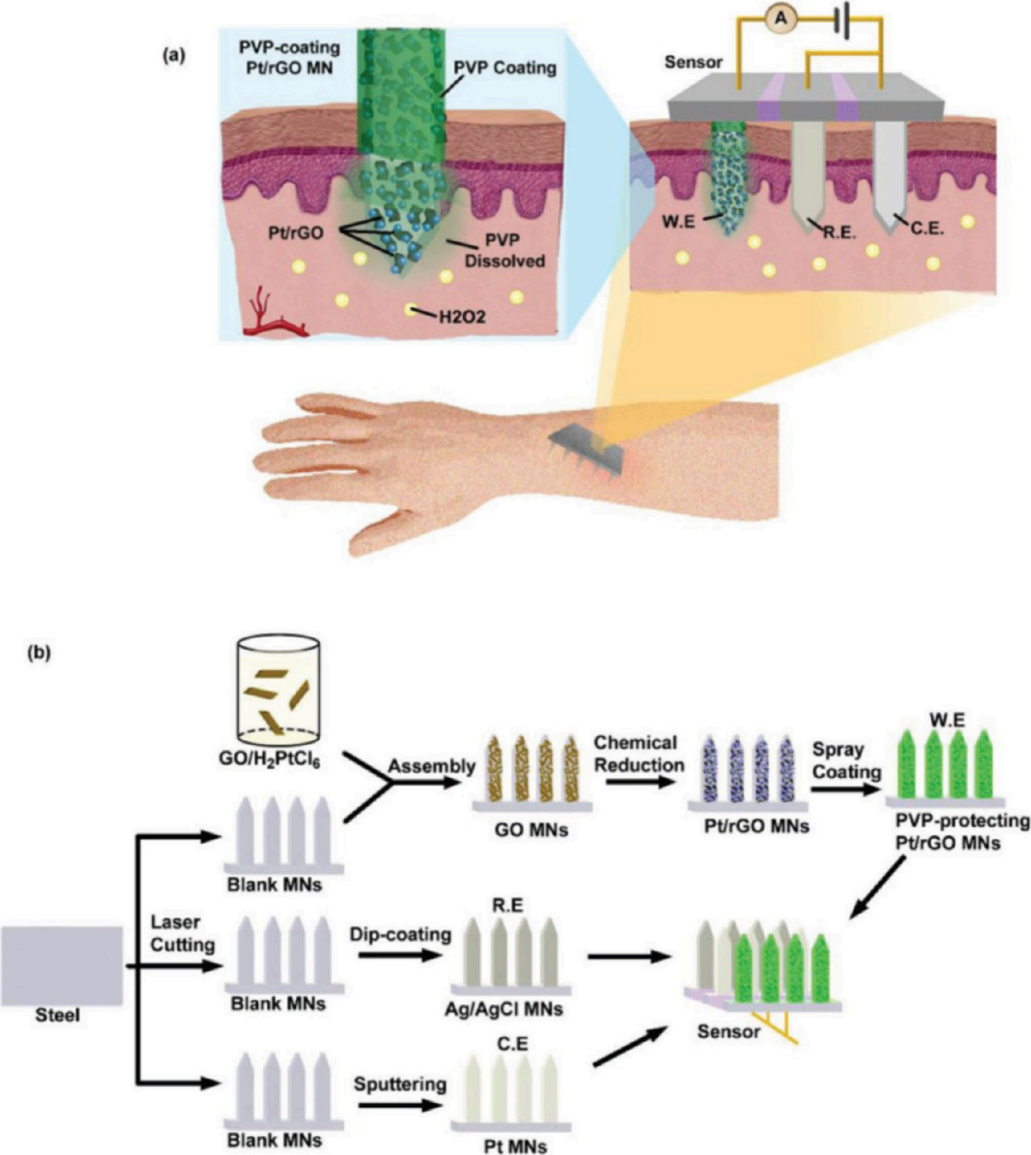
Transdermal electrochemical biosensor with Pt/rGO microneedles (MNs) for H_2_O_2_ detection. (a) Demonstration of the transdermal H_2_O_2_ electrochemical biosensor with Pt/rGO on the conductive microneedle patch for H_2_O_2_ sensing. (b) Processing of the PVP-protecting Pt/rGO microneedle patch as a transdermal H_2_O_2_ electrochemical biosensor. Reproduced with permission from ref (102). Copyright 2019, Wiley.

Portable biosensors can enable swift and timely identification of various human diseases or health conditions, offering a promising tool for early detection and intervention. Numerous biosensors, utilizing nanomaterials, have been developed for sensitive detection of biomolecules in laboratory settings. However, the translation of these biosensors for real-time monitoring within the body has seen limited success. The integration of nanostructured materials onto microneedle surfaces for transdermal electrodes remains an important issue for in vivo applications. Jin et al. developed a transdermal electrochemical biosensor for detecting hydrogen peroxide (H_2_O_2_). This biosensor utilizes a microneedle patch coated with a hybrid material consisting of platinum (Pt) nanoparticles and rGO on its surface (Pt/rGO microneedles). This hybrid material serves as the active component for detecting hydrogen peroxide (H_2_O_2_). The Pt/rGO sensing component is applied to the body using a microneedle patch, with a hydrophile polymer layer protecting the nanostructures from mechanical friction during insertion. Once inserted, the polymer layer dissolves in interstitial fluid, exposing the Pt/rGO nanostructures for in vivo H_2_O_2_ detection. The Pt/rGO hybrid noticeably enhances the sensitivity of the microneedle electrode, whereas the protective polymer coating preserves the sensitivity by preventing damage during insertion. The preparation process of the Pt/rGO microneedle-based biosensor is shown, involving laser microetching on a stainless-steel substrate to create a conductive metal microneedle patch with a thickness of 100 m (Figure 17).^102^ Biodegradable microneedle technology offers a promising solution for delivering medication through the skin, providing a sustained release of drugs directly to the targeted area, while minimizing pain and tissue damage. However, to ensure widespread adoption, it is crucial to evaluate the toxicity of microneedle components. Therefore, thorough toxicological assessments are necessary to develop safe and effective formulations for clinical use. This requires the design of polymeric Microneedles that are biodegradable, biocompatible, and nontoxic. Chitosan (CS), a biocompatible and biodegradable derivative of chitin, is an ideal material for this purpose. With its semicrystalline structure and Various medical uses, such as tissue regeneration, agriculture, as well as drug delivery, CS is encouraging for TDD demands. The mechanical weakness of CS can be overcome by adding nanofillers, and its functional groups allow for combination with other polymeric systems.

**Figure 18:**
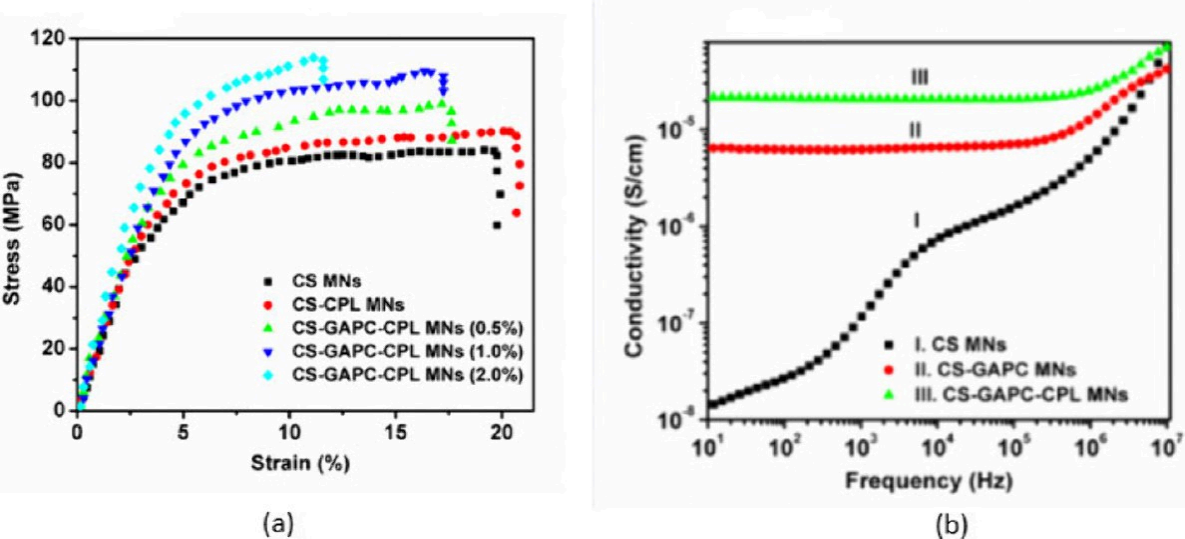
Mechanical strength and conductivity in graphene-assembled porous carbon (GAPC) microneedles (MNs). (a) Stress–strain curve of Chitosan (CS) microneedles, Chitosan/Cephalexin (CSCPL) microneedles, and their distinct ratios of GAPC. (b) CS-GAPC microneedles and CS- GAPC-CPL microneedles versus frequency, depicting the improvement of the conductivity when GAPC is included in CS microneedles. Reproduced with permission from ref (103). Copyright 2019, Wiley.

Researchers have incorporated graphene-assembled porous carbon (GAPC) into a polymer complex, leveraging graphene’s electrical, optical, thermal, and magnetic properties, as well as its high aspect ratio. A nanocomposite system consisting of CS and GAPC shows promise for drug delivery applications. This nanocomposite is used to create an array of microneedles for drug encapsulation and release studies, using Cephalexin (CPL) as a model drug. The mechanical strength, in vivo animal studies, antimicrobial properties, and toxicological safety of microneedles are evaluated. The results demonstrate the potential of microneedles to penetrate skin layers and dispense drugs effectively, proposing a smart nanocomposite system for controlled drug release (Figure 18).^103^

**Figure 19:**
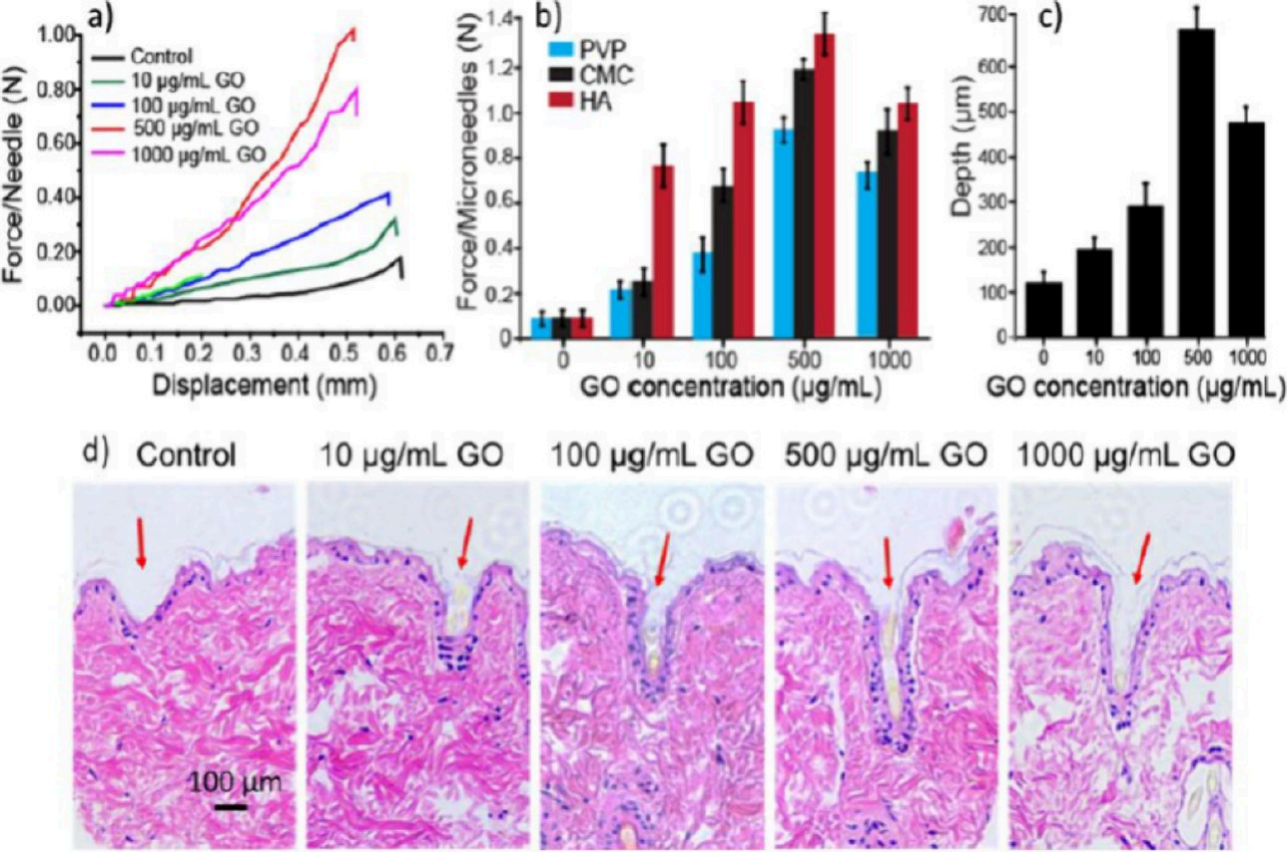
Mechanical properties and skin insertion analysis of PVP-based microneedles with graphene oxide integration. (a) Changes in force over displacement of PVP microneedles. (b) Microneedles made of PVP, CMC, and HA with various graphene oxide ratios. (c) Skin insertion depth of PVP microneedles. (d) Microscope images of skin cross sections after treatment with PVP microneedles. The microneedles insertion places are shown by red arrows. Reproduced with permission from ref (13). Copyright 2019, American Chemical Society.

To date, researchers have investigated many innovative materials, including polymers and composites, for the advancement of drug-polymer magnetic nanocomposites (DP microneedles). Adding small amounts (1–2%) of graphene-based materials, like rGO or graphene quantum dots (GQDs) into polymers has led to the development of novel properties and enhanced performance in these composite materials. This integration has led to improved physicomechanical properties and controlled dispensing of large-molecule medicines in response to electrical stimulation. Nonetheless, the remarkable mechanical characteristics of GO, including its flexibility and rigidity, have yet to be fully utilized in clinical applications, presenting an opportunity for further exploration and development. Chen et al. have demonstrated that many of the challenges associated with DP microneedles formulations can be mitigated by incorporating 0.5 wt. Their study combines in vitro along with in vivo tests to highlight the pioneering functions that GO contributes to DP microneedles. These advanced functions include improved water resistance, antibacterial and anti-inflammatory properties, light-activated drug release. These enhanced properties broaden the selection of suitable polymers for microneedles fabrication, providing numerous advantages for clinical applications and expanding the potential uses of microneedles technology (Figure 19).^13^

## 10 Biomedical Applications of Graphene-Based Microneedles

### 10.1 Release Mechanism

**Figure 20:**
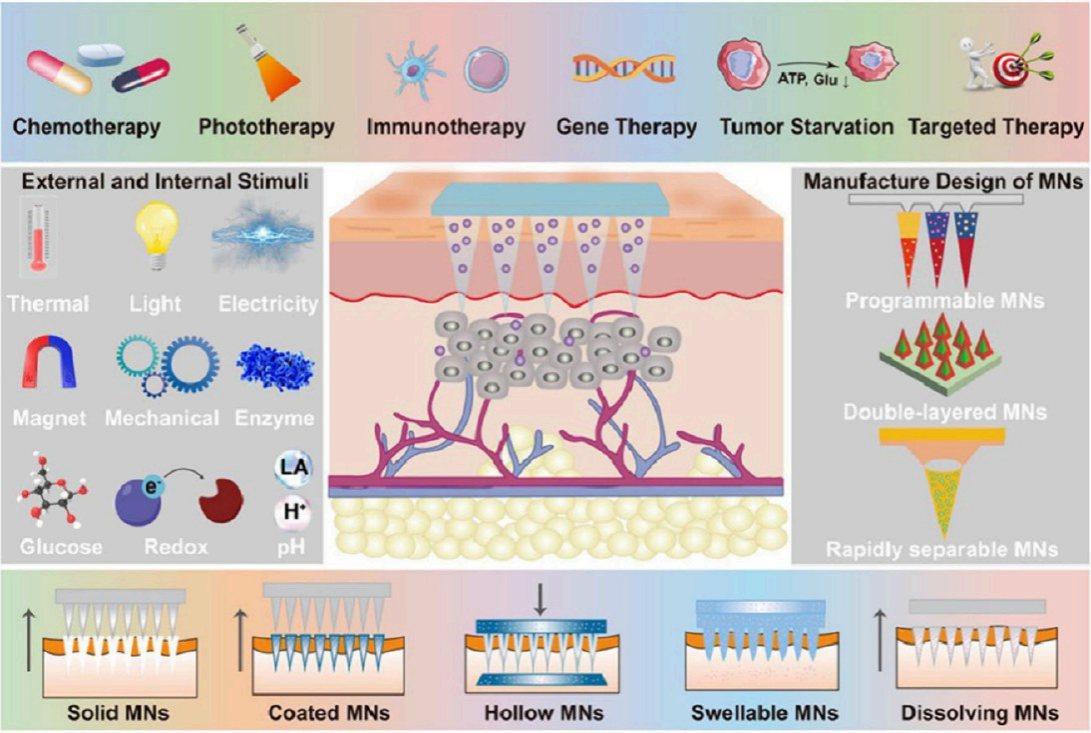
Trigger release mechanisms of microneedle-based systems for stimulation-responsive cancer therapy, showcasing the integration of diverse external and internal stimuli, manufacturing innovations, and specialized designs for targeted, controlled drug delivery. Reproduced with permission from ref (105). Copyright 2023, Elsevier.

The study of stimuli-responsive polymer microneedles has gained attention, and they can be used for drug delivery in specific times and spaces based on different patients’ needs. The microneedles can react to external environmental triggers namely light, electric fields, magnetic fields, temperature, and mechanical stress. They are categorized into different types, including light-responsive, electricity-responsive, magnetism-responsive, thermal-responsive, and mechanical-force-responsive microneedles. These responsive microneedles systems aim to achieve targeted transdermal drug delivery and improve drug efficacy.^104^Figure 20 shows the innovative use of microneedles in cancer therapy, portraying their unique ability to enable precise and localized drug delivery. Microneedles are engineered to respond to various external (thermal, light, magnetic, and electrical) and internal (pH, enzymatic, glucose, and redox) stimuli, ensuring tailored and on-demand therapeutic responses. The manufacturing approaches of Microneedles such as programmable, double-layered, and rapidly separable further enhance their versatility. The lower section highlights the structural types of microneedles, including solid, coated, hollow, swellable, and dissolvable variants, each offering distinct release profiles. These advancements make microneedles a transformative technology, combining minimally invasive delivery with high therapeutic precision to integrate multiple cancer treatments such as phototherapy, immunotherapy, and gene therapy.^105^

### 10.2 Thermal and pH Responsive Microneedles

Microneedles that respond to temperature changes utilize the phase transition properties of thermosensitive materials to regulate drug release triggered by body temperature, caused by various health conditions. Researchers have explored the application of heat-sensitive substances to control the separation of the microneedles tip from its substrate, enabling sustained drug release. In one study, nanoparticles loaded with a DNA vaccine and coated with an immune-stimulating agent were encapsulated at the microneedle tip, with a temperature-sensitive layer Poly(*N*-isopropylacrylamide) (PNIPAM) added to facilitate separation from the substrate.^106^ When applied to the skin, the tip of microneedle is retained in the dermis layer for prolonged DNA vaccine release by cooling the skin to 14–16 °C, leading to a robust and durable antiviral immune response. The microneedles demonstrated stable storage of the DNA vaccine at room temperature for up to 30 days and effective temperature-controlled separation of the tip from the substrate. The release of drugs from thermoresponsive materials is typically controlled by the formation and breakage of hydrogen bonds or molecular aggregation and disaggregation, driven by temperature changes. This process is influenced by van der Waals or hydrophobic interactions, leading to changes in molecular structure and drug release. When molecular aggregation occurs, an imbalance in hydrophilicity and hydrophobicity triggers molecular disassembly and drug secretion in response to temperature stimuli. Various thermosensitive materials, including those with lower or higher critical solution temperatures, have been utilized for drug delivery. Examples include elastin, polypeptides, carbohydrate-based materials, Pluronic hydrogels, PNIPAM, poly(acrylic acid)-*co*-acrylamide, block copolymers based on PEG, and carbon and metal nanoparticles. In a specific study, a solid microneedle with a length of 600 μm was inserted into porcine skin for 48 h, increasing skin permeability. The microneedles were loaded with Naltrexone Hydrochloride (NTX-HCl) encapsulated in poloxamer 407 (P407), which formed a gel at ambient temperatures up to 27 °C, resulting in a steady drug release rate over 48 h. In contrast, aqueous-loaded NTX-HCl exhibited a faster and higher dispensing rate. The body’s extracellular fluids and physiological pH typically maintain a balance of around 7.4, while the intracellular pH of early endosomes and late endosomes/lysosomes is slightly acidic, ranging from 6.0 to 5.0, respectively. This pH gradient between intra- and extracellular environments can be leveraged to trigger the targeted release of loaded medicines at specific intracellular sites. However, certain pathological conditions may disrupt this balance, altering the local pH. For example, tumor microenvironments are characterized by low pH, high glutathione levels, and hypoxia, distinguishing them from healthy tissues. Similarly, chronic diseases like diabetic skin wounds and chronic wound infections can significantly alter skin surface pH due to microbial activity and metabolic changes, with reported pH values ranging from 9.0 to 6.41.^107^ The pH-responsive characteristics of Zeolitic Imidazolate Framework-8 (ZIF-8) make it an ideal candidate for treating inflammatory skin conditions, including acne and basal cell carcinoma, which exhibit distinct acidic lesions in the dermis (pH 5.0–6.5) compared to healthy dermal tissue (pH 7.5–8.0). Leveraging the pH difference, Jung et al.^108^ developed a targeted drug delivery approach for treating skin inflammation. They utilized ZIF-8 as a pH-responsive nanoparticle and loaded it with the model drug curcumin (CCM). Upon exposure to the acidic environment at the inflammation site, the ZIF-8 structure degrades, releasing the bonded CCM, enabling on-demand drug delivery and targeted skin inflammation treatment. In vitro testing using buffer solutions and pig skin demonstrated that the CCMZIF microneedles system dispensed significantly higher amounts of CCM at a pH of 5.0, mimicking the acidic environment of inflammatory skin conditions, compared to a pH of 7.4, representing normal skin conditions. This indicates that the CCMZIF microneedles system can effectively respond to pH changes and release the drug in a targeted manner, making it a promising treatment for inflammatory skin conditions. Lan et al.^109^ achieved a groundbreaking cancer treatment by using microneedles to deliver lipid-coated cisplatin nanoparticles (LCC-NPs) directly through the skin. By encapsulating cisplatin in a lipid layer, they controlled the release of the nanoparticles, minimizing harm to healthy cells and ensuring targeted delivery to the tumor site. The results showed that the LCC-NPs responded to the acidic environment of the tumor, releasing the cisplatin and effectively treating cancer locally. This approach avoids the toxic side effects associated with traditional chemotherapy methods, offering a safer and more efficient alternative for cancer treatment.

### 10.3 Light Responsive Microneedles

Light offers several advantages, including noninvasiveness, high spatial resolution, and precise temporal control, making it an attractive tool for medical applications. Light-responsive materials, which react to light, are commonly used in chemotherapy and photothermal therapy. These materials are created by incorporating photosensitive nanoparticles, such as LaB6, Pt, Au, and TiO_2_, or photochromic agents including melanin into polymeric microneedles.^110^ Near-infrared (NIR) radiation is the most frequently used electromagnetic wave for activating these materials. Light-activated microneedles respond through various mechanisms, including the use of phase-change polymers that melt and release their contents when exposed to light. These polymers can be blended with photothermal conversion agents and can adjust their melting temperature by incorporating metallic materials. For example, LaB6 nanoparticles generate heat in response to NIR irradiation, making them suitable for light-activated microneedles. Microneedles composed of LaB6 and polycaprolactone composites enable controlled drug release by melting polycaprolactone when exposed to NIR radiation. This technology has potential applications in diabetes treatment, where light-sensitive materials can regulate drug release in response to an external NIR radiation source. For example, researchers have developed hollow mesoporous SiO_2_ nanoparticles encapsulated with metformin, coated with polydopamine and lauric acid. The polydopamine acts as a photothermal conversion agent, causing the lauric acid layer (with a melting point of 44–46 °C) to melt upon NIR irradiation, releasing the metformin. This innovative approach allows for precise control over drug delivery. The mesoporous SiO_2_ structure allows for the controlled release of metformin through diffusion, with minimal release occurring without NIR laser irradiation. However, significant metformin release is achieved after five cycles of NIR laser activation.^110^ This NIR-triggered microneedles patch offers a reduced risk of hypoglycemia and demonstrates no in vivo toxicity. Additionally, light-activated prodrugs can be synthesized through the chemical coupling of photocleavable compounds, enabling the liberation of encapsulated bioactive drugs upon light irradiation. For instance, conjugating drugs like acetylsalicylic acid, ibuprofen, and ketoprofen with photoactivatable compounds like dimethylamino pyridine imparts light responsiveness, allowing the prodrug to be cleaved and released from microneedles. This approach enables precise control over drug delivery and activation.^110^ The final category of light-activated Microneedles operates by transforming light into heat, thereby increasing the surrounding temperature. A notable instance is the development of a patch containing melanin, intended for use in photothermal treatment to boost the effectiveness of cancer immunotherapy. In this approach, a patch with melanin and tumor lysate from melanoma is used for NIR PTT. When combined with NIR irradiation, the patch promotes tumor cell death, facilitating the uptake of tumor-associated antigens by dendritic cells and enhancing antitumor immune responses. Notably, a vaccine microneedles patch with NIR irradiation demonstrated magnificent tumor growth suppression compared to control groups, including those treated with a melanin-only microneedles patch, a hyaluronic acid-loaded microneedles patch with NIR irradiation or a vaccine microneedles patch without NIR irradiation. This innovative approach shows promising results for cancer treatment.^110^

### 10.4 Glucose Responsive Microneedles

Microneedles implemented into the skin interact with the dermal microcirculation, allowing real-time monitoring of metabolic changes in the biological environment. For individuals with diabetes, frequent blood glucose monitoring is crucial for timely administration of antidiabetic medications. Recent advancements have led to the development of glucose-responsive microneedles, enabling convenient and painless insulin delivery. Gu and colleagues created a pioneering closed-loop glucose-responsive microneedles patch, featuring glucose-responsive vesicles loaded with insulin and glucose oxidase (GOx).^110^ These vesicles, composed of hypoxia-sensitive 2-nitroimidazole-modified hyaluronic acid, release insulin in response to high blood glucose levels. When GOx induces hypoxia, the hydrophobic 2-nitroimidazole converts to hydrophilic 2-aminoimidazole, causing vesicle dissociation and insulin release. In type I diabetic mice, these microneedles rapidly normalized blood glucose levels and maintained normal glycemia for 4 h.

### 10.5 Enzyme Responsive Microneedles

Enzymes play a vital role in maintaining good health by regulating metabolic reactions. However, enzyme dysfunction or abnormal expression is associated with various diseases, including cancer, where tumors exhibit distinct enzyme profiles that can be targeted for diagnosis and treatment. Enzyme-responsive microneedles also show promise in antibacterial therapy, particularly in wound healing. Research has demonstrated that gelatinase is overexpressed at wound sites, making gelatinase-responsive Microneedles an effective tool for antimicrobial treatment. For instance, Liu et al.^106^ developed a degradable microneedles patch that releases antibacterial peptides in response to elevated gelatinase levels, promoting wound healing and exhibiting significant therapeutic effects in mouse models. The advantage of enzyme-responsive microneedles lies in their ability to exploit differences in enzyme expression between pathological and normal environments, enabling targeted treatment while minimizing toxicity. However, achieving controlled drug release solely through enzyme response can be challenging, and combining enzyme response with other stimuli can enhance drug utilization efficiency.

### 10.6 Derivative Graphene Microneedles

Previous research has shown that Porous Graphene (PG) exhibits higher electrochemical activity and electron transfer kinetics compared to Porous Carbon (PC), likely due to its increased edge defects and functional groups. However, this also leads to reduced stability in PG/ISE electrodes. Therefore, PC electrodes, with their superior stability, were selected for integration into a microneedle ISE sensor. SEM images reveal that the ISE membrane fully penetrates the PC’s porous structure, conformally coating the material and contributing to the sensor’s exceptional stability. The three-dimensional nature of the sensor enhances sensitivity by increasing the surface area, making it suitable for point-of-care diagnostic applications where accurate electrolyte level determination is crucial. A novel transdermal microneedles sensor has been developed for potassium detection by combining a hollow microneedle with a microfluidic chip. This innovative setup enables fluid extraction and analysis using a downstream solid-state ISE. Researchers evaluated both 3D porous carbon and 3D porous graphene electrodes, fabricated with interference lithography, as potential solid-state transducers for ISEs. The porous carbon potassium ISEs demonstrated superior performance, stability, and selectivity compared to porous graphene ISEs, accurately measuring potassium levels within physiological concentrations despite interference from other ions. This groundbreaking microfluidic/microneedles platform holds promise for various medical applications, offering a potential breakthrough in potassium monitoring and detection.^69^ In addition to enhancing mechanical and electrical characteristics, incorporating graphene nanoparticles into a nanocomposite improves drug release behavior while maintaining the biodegradability of the polymer, surpassing its polymer counterpart. Previous research has demonstrated that GO and rGO can effectively bind to therapeutics and be released from chitosan, resulting in significantly higher drug release and pH-sensitive delivery. Furthermore, the integration of quantum dots (QDs) enables real-time tracking of drug diffusion in the body after dispensing from a microneedle array under fluorescent light. QDs, such as cadmium telluride (CdTe) or cadmium sulfide (CdS), retain their photoluminescent properties when encapsulated in polymers, making them suitable for bioimaging applications, including cell tracking and various devices like X-ray scintillation detectors and optoelectronic devices. Conventional QDs have limited biomedical applications due to their toxicity, particularly cadmium-based QDs, which are cytotoxic and damage DNA. In contrast, carbon-based QDs exhibit low toxicity in vivo, making them suitable for biomedical applications, especially when coated with a biocompatible polymer or used at low concentrations. Given their favorable properties, they proposed that chitosan-GQD nanocomposites hold promise for application in microneedles arrays for iontophoresis and tracked transdermal drug delivery, offering a novel and safer approach for biomedical applications. This research direction has yet to be explored, presenting an opportunity to develop innovative and biocompatible QD-based nanocomposites for transdermal drug delivery. GQDs were synthesized through hydrothermal reduction of GO, resulting in particles with a diameter of 50–55 nm and a height of ∼1.5 nm. These GQDs exhibited photoluminescent properties and showed minimal cytotoxicity to adipose-derived mesenchymal stem cells (MSCs), making them suitable for fluorescent imaging of cells. Chitosan-GQD nanocomposites were prepared using solution casting, leading to a 7.9-fold increase in electrical conductivity compared to pristine chitosan. The biodegradation rate of the nanocomposite remained similar to pristine chitosan after 28 days. Additionally, the incorporation of 1 wt % GQDs enhanced the ultimate tensile strength of chitosan from 62.5 to 84.8 MPa and increased the elongation to break from 15.5% to 21.2%, while maintaining a consistent Young’s modulus. The incorporation of graphene quantum dots (GQD) did not hinder the biodegradation of chitosan, with both pristine chitosan and the nanocomposite exhibiting similar mass remaining after 28 days. Although the nanocomposite initially showed a faster biodegradation rate, it eventually leveled off. When formed into microneedles, the chitosan-1 wt % GQD nanocomposite demonstrates the potential for transdermal drug delivery. The microneedles arrays maintained their structural integrity during insertion into the body and released the painkiller lidocaine hydrochloride more efficiently than pristine chitosan microneedles, with a 68.3% release rate compared to 57.4%. This enhanced drug release suggests that the chitosan-GQD nanocomposite microneedles could be an auspicious platform for transdermal drug delivery. The nanocomposite microneedles demonstrated the ability to release large molecular weight drugs, such as bovine serum albumin, in response to electrical stimulation. Notably, the iontophoresis-effect microneedles showed a significant increase in drug release, from 7.6% to 94.5% of the available drug, over 24 h. Additionally, the stability of GQDs and GQD-LH (GQDs with lipo hydrazide) was evaluated in various solutions, including distilled water, PBS, and fetal calf serum. The results showed that GQDs and GQD-LH remained stable over 24 h, with GQD-LH exhibiting extended stability in fetal bovine serum for up to 10 days because of the electrostatic repulsion effect imparted by lipo hydrazide.^8^ Recent advancements in electrochemical biosensors utilize nanomaterials like graphene and rGO due to their exceptional charge transport properties, large surface area. These materials have been combined with metal nanoparticles to create hybrid nanocomposites, resulting in highly sensitive electrodes. For example, graphene/Pt nanoparticle hybrids have been used to develop various biosensors. However, most existing biosensors require extracted biofluids to be transferred onto the sensor chip for in vitro detection, limiting real-time in vivo sensing capabilities. To address this, researchers have integrated biosensors onto metal needles for in vivo penetration, but this method poses risks to wounds and infections, restricting long-term sensing applications.^102^ The loading efficiency of PVA microneedle patches with varying concentrations of GO was measured. Patches with 0.8% and 1.6% GO had high loading efficiencies (>50%), likely due to GO’s porous structure and electrostatic interactions with VEGF. In contrast, patches with 0.2% and 0.4% GO had lower loading efficiencies (<50%). The drug release rate was also affected by GO concentration. Neat PVA patches released 66.4% of VEGF within 24 h, whereas GO-containing patches released less than 20.5% in the same time frame. At 72 h, GO-containing patches still had lower release amounts (<40%), indicating prolonged release times. The 0.8% GO-PVA patch showed the highest release efficiency (18% of total drug load), while the 1.6% GO-PVA patch had lower release efficiency (10%) due to potential trapping effects and reduced porosity. Therefore, 0.8% GO-PVA hydrogel was selected for microneedle patches to optimize VEGF utilization and achieve sustained release.^111^

## 11 Future Directions

The field of graphene-based microneedles for biomedical applications holds great promise for advancing medical technologies. To fully realize this potential, several key areas of research and development must be addressed. One critical aspect is the improvement of manufacturing processes. Efforts should focus on developing more efficient and cost-effective fabrication methods for graphene-based microneedles. This includes exploring novel synthesis techniques, enhancing surface modification approaches, and integrating these microneedles with existing medical platforms. Additionally, there is a need for further research in design and structural engineering. Optimizing the physical and mechanical properties of Microneedles through careful design considerations, such as shape, dimension, and porosity, is essential for tailoring these devices to specific medical applications. Comprehensive studies on the safety and biocompatibility of graphene-based microneedles at the cellular, molecular, and tissue levels are necessary to ensure their suitability for clinical use. Furthermore, researchers should investigate their emerging applications in smart drug delivery, biosensing, and tissue engineering fields. Exploring new avenues for these devices can expand their potential impact on healthcare. Lastly, integrating graphene-based microneedles with cutting-edge technologies, such as electronic sensors and intelligent control systems, can lead to the development of multifunctional platforms with enhanced capabilities. Continued research and development in the aforementioned areas are essential to unlock the full potential of these innovative devices in improving healthcare outcomes.

## 12 Conclusions

We have highlighted an overview of the significant advancements in the field of Microneedles, particularly showing the prospects of microneedles devices based on graphene in healthcare applications. The fundamental principles of microneedles operation, including their fabrication techniques, classification, and diverse applications, have been succinctly explained. We also emphasized the importance of constituent materials used in different microneedles categories, concentrating on the exceptional properties of graphene and its suitability for integration with microneedles technology. Graphene has shown great promise in the medical field, demonstrating excellent compatibility and performance enhancements when incorporated into microneedle systems. The production methods, associated challenges, common applications, and other relevant aspects of this topic were examined and discussed. We have highlighted the improvements in manufacturing processes, structural engineering, biocompatibility evaluation, and the exploration of emerging applications for future research and development. The insights provided in this study will hopefully serve as a valuable resource for researchers, clinicians, and industry professionals working toward the further development and implementation of graphene-based microneedles in the biomedical field.

## References

[ref1] Wang M.; Hu L.; Xu C. Recent Advances in the Design of Polymeric Microneedles for Transdermal Drug Delivery and Biosensing. Lab Chip 2017, 17 (8), 1373–1387. 10.1039/C7LC00016B. 28352876

[ref2] Ingrole R. S.; Azizoglu E.; Dul M.; Birchall J. C.; Gill H. S.; Prausnitz M. R. Trends of Microneedle Technology in the Scientific Literature, Patents, Clinical Trials and Internet Activity. Biomaterials 2021, 267, 120491 10.1016/j.biomaterials.2020.120491. 33217629 PMC8042615

[ref3] Ma G.; Wu C. Microneedle, Bio-Microneedle and Bio-Inspired Microneedle: A Review. J. Controlled Release 2017, 251, 11–23. 10.1016/j.jconrel.2017.02.011. 28215667

[ref4] Kim H.; Yoon H.; Sharifuzzaman M.; Park J.; Kim D.; Park J. Skin-Attachable and Implantable Polymer Microneedle Biosensor for Continuous Glucose Monitoring. In 2020 IEEE 33rd International Conference on Micro Electro Mechanical Systems (MEMS); IEEE, 2020; pp 404–407, 10.1109/MEMS46641.2020.9056159.

[ref5] Zhao J.; Duan W.; Liu X.; Xi F.; Wu J. Microneedle Patch Integrated with Porous Silicon Confined Dual Nanozymes for Synergistic and Hyperthermia-enhanced Nanocatalytic Ferroptosis Treatment of Melanoma. Adv. Funct. Mater. 2023, 33 (47), 2308183 10.1002/adfm.202308183.

[ref6] Faraji Rad Z.; Prewett P. D.; Davies G. J. An Overview of Microneedle Applications, Materials, and Fabrication Methods. Beilstein journal of nanotechnology 2021, 12 (1), 1034–1046. 10.3762/bjnano.12.77. 34621614 PMC8450954

[ref7] Amarnani R.; Shende P. Microneedles in Diagnostic, Treatment and Theranostics: An Advancement in Minimally-Invasive Delivery System. Biomed. Microdevices 2022, 24 (1), 4 10.1007/s10544-021-00604-w. PMC8651504 34878589

[ref8] Justin R.; Chen B. Multifunctional Chitosan–Magnetic Graphene Quantum Dot Nanocomposites for the Release of Therapeutics from Detachable and Non-Detachable Biodegradable Microneedle Arrays. Interface Focus 2018, 8 (3), 20170055 10.1098/rsfs.2017.0055. 29696087 PMC5915657

[ref9] Mdanda S.; Ubanako P.; Kondiah P. P.; Kumar P.; Choonara Y. E. Recent Advances in Microneedle Platforms for Transdermal Drug Delivery Technologies. Polymers 2021, 13 (15), 2405 10.3390/polym13152405. 34372008 PMC8348894

[ref10] Howells O.; Rajendran N.; Mcintyre S.; Amini-Asl S.; Henri P.; Liu Y.; Guy O.; Cass A. E. G.; Morris M. C.; Sharma S. Microneedle Array-Based Platforms for Future Theranostic Applications. ChemBioChem. 2019, 20 (17), 2198–2202. 10.1002/cbic.201900112. 30897259

[ref11] Dafe V. N.; Hatwar P. R.; Bakal R. L.; Kubde J. A.; Jumde K. S.; Dafe V. N.; Hatwar P. R.; Bakal R. L.; Kubde J. A.; Jumde K. S. Transdermal Insulin Delivery via Microneedle Technology, Patches, and Pumps Offers a Promising Alternative to Traditional Subcutaneous Injections for Diabetes Management. GSC Biological and Pharmaceutical Sciences 2024, 29 (1), 233–242. 10.30574/gscbps.2024.29.1.0372.

[ref12] Chiulan I.; Voicu Ş. I.; Batalu D. The Use of Graphene and Its Derivatives for the Development of Polymer Matrix Composites by Stereolithographic 3D Printing. Applied Sciences 2022, 12 (7), 3521 10.3390/app12073521.

[ref13] Chen Y.; Yang Y.; Xian Y.; Singh P.; Feng J.; Cui S.; Carrier A.; Oakes K.; Luan T.; Zhang X. Multifunctional Graphene-Oxide-Reinforced Dissolvable Polymeric Microneedles for Transdermal Drug Delivery. ACS Appl. Mater. Interfaces 2020, 12 (1), 352–360. 10.1021/acsami.9b19518. 31825580

[ref14] Tabish T. A.; Abbas A.; Narayan R. J. Graphene Nanocomposites for Transdermal Biosensing. Wiley Interdisciplinary Reviews: Nanomedicine and Nanobiotechnology 2021, 13 ( (4), ), 10.1002/wnan.1699. 33480118

[ref15] Farjadian F.; Abbaspour S.; Sadatlu M. A. A.; Mirkiani S.; Ghasemi A.; Hoseini-Ghahfarokhi M.; Mozaffari N.; Karimi M.; Hamblin M. R. Recent Developments in Graphene and Graphene Oxide: Properties, Synthesis, and Modifications: A Review. ChemistrySelect 2020, 5 (33), 10200–10219. 10.1002/slct.202002501.

[ref16] Kim Y.-C.; Park J.-H.; Prausnitz M. R. Microneedles for Drug and Vaccine Delivery. Advanced drug delivery reviews 2012, 64 (14), 1547–1568. 10.1016/j.addr.2012.04.005. 22575858 PMC3419303

[ref17] Yadav P. R.; Munni M. N.; Campbell L.; Mostofa G.; Dobson L.; Shittu M.; Pattanayek S. K.; Uddin M. J.; Das D. B. Translation of Polymeric Microneedles for Treatment of Human Diseases: Recent Trends, Progress, and Challenges. Pharmaceutics 2021, 13 (8), 1132 10.3390/pharmaceutics13081132. 34452093 PMC8401662

[ref18] Aldawood F. K.; Parupelli S. K.; Andar A.; Desai S. 3D Printing of Biodegradable Polymeric Microneedles for Transdermal Drug Delivery Applications. Pharmaceutics 2024, 16 (2), 237 10.3390/pharmaceutics16020237. 38399291 PMC10893432

[ref19] Aldawood F. K.; Andar A.; Desai S. A Comprehensive Review of Microneedles: Types, Materials, Processes, Characterizations and Applications. Polymers 2021, 13 (16), 2815 10.3390/polym13162815. 34451353 PMC8400269

[ref20] Chudzińska J.; Wawrzyńczak A.; Feliczak-Guzik A. Microneedles Based on a Biodegradable Polymer—Hyaluronic Acid. Polymers 2024, 16 (10), 1396 10.3390/polym16101396. 38794589 PMC11124840

[ref21] Starlin Chellathurai M.; Mahmood S.; Mohamed Sofian Z.; Wan Hee C.; Sundarapandian R.; Ahamed H. N.; Kandasamy C. S.; Hilles A. R.; Hashim N. M.; Janakiraman A. K. Biodegradable Polymeric Insulin Microneedles–a Design and Materials Perspective Review. Drug Delivery 2024, 31 (1), 2296350 10.1080/10717544.2023.2296350. 38147499 PMC10763835

[ref22] Mutlu M. E.; Ulag S.; Sengor M.; Daglılar S.; Narayan R.; Gunduz O. Electrosprayed Collagen/Gentamicin Nanoparticles Coated Microneedle Patches for Skin Treatment. Mater. Lett. 2021, 305, 130844 10.1016/j.matlet.2021.130844.

[ref23] Yang J.; Liu X.; Fu Y.; Song Y. Recent Advances of Microneedles for Biomedical Applications: Drug Delivery and Beyond. Acta Pharmaceutica Sinica B 2019, 9 (3), 469–483. 10.1016/j.apsb.2019.03.007. 31193810 PMC6543086

[ref24] Damiri F.; Kommineni N.; Ebhodaghe S. O.; Bulusu R.; Jyothi V. G. S.; Sayed A. A.; Awaji A. A.; Germoush M. O.; Al-Malky H. S.; Nasrullah M. Z. Microneedle-Based Natural Polysaccharide for Drug Delivery Systems (DDS): Progress and Challenges. Pharmaceuticals 2022, 15 (2), 190 10.3390/ph15020190. 35215302 PMC8875238

[ref25] Ali M.; Namjoshi S.; Benson H. A.; Mohammed Y.; Kumeria T. Dissolvable Polymer Microneedles for Drug Delivery and Diagnostics. J. Controlled Release 2022, 347, 561–589. 10.1016/j.jconrel.2022.04.043. 35525331

[ref26] Cao Y.; Tao Y.; Zhou Y.; Gui S. Development of Sinomenine Hydrochloride-Loaded Polyvinylalcohol/Maltose Microneedle for Transdermal Delivery. Journal of Drug Delivery Science and Technology 2016, 35, 1–7. 10.1016/j.jddst.2016.06.007.

[ref27] Chen C.; Fu X.; Fan W.; Ma T. Synthesis and Electrochemical Properties of Graphene Oxide/Acenaphthenequinone Composite. Nano 2015, 10 (02), 1550023 10.1142/S179329201550023X.

[ref28] Skákalová V.; Kaiser A. B. Graphene: Properties, Preparation, Characterization and Applications; Woodhead Publishing, 2021.

[ref29] Saeed M.; Alshammari Y.; Majeed S. A.; Al-Nasrallah E. Chemical Vapour Deposition of Graphene—Synthesis, Characterisation, and Applications: A Review. Molecules 2020, 25 (17), 3856 10.3390/molecules25173856. 32854226 PMC7503287

[ref30] Farmanbordar-Ghadikolaei N.; Kowsari E.; Taromi F. A.; Vatanpour V.; Abdollahi H. High-Performance Functionalized Graphene Oxide Reinforced Hyperbranched Polymer Nanocomposites for Catalytic Hydrolysis of a Chiral Ester in Water. React. Funct. Polym. 2022, 173, 105218 10.1016/j.reactfunctpolym.2022.105218.

[ref31] Tadyszak K.; Wychowaniec J. K.; Litowczenko J. Biomedical Applications of Graphene-Based Structures. Nanomaterials 2018, 8 (11), 944 10.3390/nano8110944. 30453490 PMC6267346

[ref32] Priyadarsini S.; Mohanty S.; Mukherjee S.; Basu S.; Mishra M. Graphene and Graphene Oxide as Nanomaterials for Medicine and Biology Application. Journal of Nanostructure in Chemistry 2018, 8, 123–137. 10.1007/s40097-018-0265-6.

[ref33] Pang B.; Lin S.; Shi Y.; Wang Y.; Chen Y.; Ma S.; Feng J.; Zhang C.; Yu L.; Dong L. Synthesis of CoFe2O4/Graphene Composite as a Novel Counter Electrode for High Performance Dye-Sensitized Solar Cells. Electrochim. Acta 2019, 297, 70–76. 10.1016/j.electacta.2018.11.170.

[ref34] Najafi M.; Zahid M.; Ceseracciu L.; Safarpour M.; Athanassiou A.; Bayer I. S. Polylactic Acid-Graphene Emulsion Ink Based Conductive Cotton Fabrics. Journal of Materials Research and Technology 2022, 18, 5197–5211. 10.1016/j.jmrt.2022.04.119.

[ref35] Kim S.-W.; Kwon S.-N.; Na S.-I. Stretchable and Electrically Conductive Polyurethane-Silver/Graphene Composite Fibers Prepared by Wet-Spinning Process. Composites Part B: Engineering 2019, 167, 573–581. 10.1016/j.compositesb.2019.03.035.

[ref36] Janković A.; Eraković S.; Vukašinović-Sekulić M.; Mišković-Stanković V.; Park S. J.; Rhee K. Y. Graphene-Based Antibacterial Composite Coatings Electrodeposited on Titanium for Biomedical Applications. Prog. Org. Coat. 2015, 83, 1–10. 10.1016/j.porgcoat.2015.01.019.

[ref37] Khakpour E.; Salehi S.; Naghib S. M.; Ghorbanzadeh S.; Zhang W. Graphene-Based Nanomaterials for Stimuli-Sensitive Controlled Delivery of Therapeutic Molecules. Front. Bioeng. Biotechnol. 2023, 11, 10.3389/fbioe.2023.1129768. PMC9947473 36845181

[ref38] Chung C.; Kim Y.-K.; Shin D.; Ryoo S.-R.; Hong B. H.; Min D.-H. Biomedical Applications of Graphene and Graphene Oxide. Accounts of Chemical Research 2013, 46 (10), 2211–2224. 10.1021/ar300159f. 23480658

[ref39] Geim A. K.; Novoselov K. S. The Rise of Graphene. Nat. Mater. 2007, 6 (3), 183–191. 10.1038/nmat1849. 17330084

[ref40] Adetayo A.; Runsewe D. Synthesis and Fabrication of Graphene and Graphene Oxide: A Review. Open Journal of Composite Materials 2019, 9 (02), 207 10.4236/ojcm.2019.92012.

[ref41] Dasari Shareena T. P.; McShan D.; Dasmahapatra A. K.; Tchounwou P. B. A Review on Graphene-Based Nanomaterials in Biomedical Applications and Risks in Environment and Health. Nano-Micro Letters 2018, 10, 1–34. 10.1007/s40820-018-0206-4. 30079344 PMC6075845

[ref42] Zhang B.; Wang Y.; Zhai G. Biomedical Applications of the Graphene-Based Materials. Materials Science and Engineering: C 2016, 61, 953–964. 10.1016/j.msec.2015.12.073. 26838925

[ref43] Güler Ö.; Bağcı N. A Short Review on Mechanical Properties of Graphene Reinforced Metal Matrix Composites. Journal of Materials Research and Technology 2020, 9 (3), 6808–6833. 10.1016/j.jmrt.2020.01.077.

[ref44] Zhen Z.; Zhu H. 1 - Structure and Properties of Graphene. In Graphene; Zhu H., Xu Z., Xie D., Fang Y., Eds.; Academic Press, 2018; pp 1–12, 10.1016/B978-0-12-812651-6.00001-X.

[ref45] Liu D.; Zhang S.; Zhang E.; Ma N.; Chen H. Anomalous Valley Magnetic Moment of Graphene. Europhys. Lett. 2010, 89 (3), 37002 10.1209/0295-5075/89/37002.

[ref46] Stolyarova E.; Rim K. T.; Ryu S.; Maultzsch J.; Kim P.; Brus L. E.; Heinz T. F.; Hybertsen M. S.; Flynn G. W. High-Resolution Scanning Tunneling Microscopy Imaging of Mesoscopic Graphene Sheets on an Insulating Surface. Proc. Natl. Acad. Sci. U. S. A. 2007, 104 (22), 9209–9212. 10.1073/pnas.0703337104. 17517635 PMC1874226

[ref47] Son Y.-W.; Cohen M. L.; Louie S. G. Energy Gaps in Graphene Nanoribbons. Physical review letters 2006, 97 (21), 216803 10.1103/PhysRevLett.97.216803. 17155765

[ref48] Ye T.; Yang Y.; Bai J.; Wu F.-Y.; Zhang L.; Meng L.-Y.; Lan Y. The Mechanical, Optical, and Thermal Properties of Graphene Influencing Its Pre-Clinical Use in Treating Neurological Diseases. Frontiers in Neuroscience 2023, 17, 1162493 10.3389/fnins.2023.1162493. 37360172 PMC10288862

[ref49] Mbayachi V. B.; Ndayiragije E.; Sammani T.; Taj S.; Mbuta E. R. Graphene Synthesis, Characterization and Its Applications: A Review. Results in Chemistry 2021, 3, 100163 10.1016/j.rechem.2021.100163.

[ref50] Syama S.; Mohanan P. V. Comprehensive Application of Graphene: Emphasis on Biomedical Concerns. Nano-micro letters 2019, 11, 1–31. 10.1007/s40820-019-0237-5. 34137957 PMC7770934

[ref51] Kośla K.; Olejnik M.; Olszewska K. Preparation and Properties of Composite Materials Containing Graphene Structures and Their Applicability in Personal Protective Equipment: A Review. Reviews on Advanced Materials Science 2020, 59 (1), 215–242. 10.1515/rams-2020-0025.

[ref52] Kumar P.; Huo P.; Zhang R.; Liu B. Antibacterial Properties of Graphene-Based Nanomaterials. Nanomaterials 2019, 9 (5), 737 10.3390/nano9050737. 31086043 PMC6567318

[ref53] Rashidzadeh H.; Tabatabaei Rezaei S. J.; Adyani S. M.; Abazari M.; Rahamooz Haghighi S.; Abdollahi H.; Ramazani A. Recent Advances in Targeting Malaria with Nanotechnology-Based Drug Carriers. Pharm. Dev. Technol. 2021, 26 (8), 807–823. 10.1080/10837450.2021.1948568. 34190000

[ref54] Reina G.; González-Domínguez J. M.; Criado A.; Vázquez E.; Bianco A.; Prato M. Promises, Facts and Challenges for Graphene in Biomedical Applications. Chem. Soc. Rev. 2017, 46 (15), 4400–4416. 10.1039/C7CS00363C. 28722038

[ref55] Mao H. Y.; Laurent S.; Chen W.; Akhavan O.; Imani M.; Ashkarran A. A.; Mahmoudi M. Graphene: Promises, Facts, Opportunities, and Challenges in Nanomedicine. Chem. Rev. 2013, 113 (5), 3407–3424. 10.1021/cr300335p. 23452512

[ref56] Gu H.; Tang H.; Xiong P.; Zhou Z. Biomarkers-Based Biosensing and Bioimaging with Graphene for Cancer Diagnosis. Nanomaterials 2019, 9 (1), 130 10.3390/nano9010130. 30669634 PMC6358776

[ref57] Zhang B. Y.; Liu T.; Meng B.; Li X.; Liang G.; Hu X.; Wang Q. J. Broadband High Photoresponse from Pure Monolayer Graphene Photodetector. Nat. Commun. 2013, 4 (1), 1811 10.1038/ncomms2830. 23651999

[ref58] Li Z.; Zhang W.; Xing F. Graphene Optical Biosensors. International journal of molecular sciences 2019, 20 (10), 2461 10.3390/ijms20102461. 31109057 PMC6567174

[ref59] Lee T.-J.; Park S.; Bhang S. H.; Yoon J.-K.; Jo I.; Jeong G.-J.; Hong B. H.; Kim B.-S. Graphene Enhances the Cardiomyogenic Differentiation of Human Embryonic Stem Cells. Biochemical and biophysical research communications 2014, 452 (1), 174–180. 10.1016/j.bbrc.2014.08.062. 25152405

[ref60] Barnes J. M.; Przybyla L.; Weaver V. M. Tissue Mechanics Regulate Brain Development, Homeostasis and Disease. Journal of cell science 2017, 130 (1), 71–82. 10.1242/jcs.191742. 28043968 PMC5394781

[ref61] Bellet P.; Gasparotto M.; Pressi S.; Fortunato A.; Scapin G.; Mba M.; Menna E.; Filippini F. Graphene-Based Scaffolds for Regenerative Medicine. Nanomaterials 2021, 11 (2), 404 10.3390/nano11020404. 33562559 PMC7914745

[ref62] Du Z.; Wang C.; Zhang R.; Wang X.; Li X. Applications of Graphene and Its Derivatives in Bone Repair: Advantages for Promoting Bone Formation and Providing Real-Time Detection, Challenges and Future Prospects. International journal of nanomedicine 2020, Volume 15, 7523–7551. 10.2147/IJN.S271917. PMC7547809 33116486

[ref63] Gurunathan S.; Kim J.-H. Synthesis, Toxicity, Biocompatibility, and Biomedical Applications of Graphene and Graphene-Related Materials. International journal of nanomedicine 2016, 1927–1945. 10.2147/IJN.S105264. 27226713 PMC4863686

[ref64] Joshi N.; Machekposhti S. A.; Narayan R. J. Evolution of Transdermal Drug Delivery Devices and Novel Microneedle Technologies: A Historical Perspective and Review. JID Innovations 2023, 3, 100225 10.1016/j.xjidi.2023.100225. 37744689 PMC10514214

[ref65] Prausnitz M. R.; Langer R. Transdermal Drug Delivery. Nature biotechnology 2008, 26 (11), 1261–1268. 10.1038/nbt.1504. PMC2700785 18997767

[ref66] Giri Nandagopal M. S.; Antony R.; Rangabhashiyam S.; Sreekumar N.; Selvaraju N. Overview of Microneedle System: A Third Generation Transdermal Drug Delivery Approach. Microsystem technologies 2014, 20, 1249–1272. 10.1007/s00542-014-2233-5.

[ref67] Al-Japairai K. A. S.; Mahmood S.; Almurisi S. H.; Venugopal J. R.; Hilles A. R.; Azmana M.; Raman S. Current Trends in Polymer Microneedle for Transdermal Drug Delivery. International journal of pharmaceutics 2020, 587, 119673 10.1016/j.ijpharm.2020.119673. 32739388 PMC7392082

[ref68] Jung J. H.; Jin S. G. Microneedle for Transdermal Drug Delivery: Current Trends and Fabrication. Journal of pharmaceutical investigation 2021, 51, 503–517. 10.1007/s40005-021-00512-4. 33686358 PMC7931162

[ref69] Miller P. R.; Xiao X.; Brener I.; Burckel D. B.; Narayan R.; Polsky R. Microneedle-based Transdermal Sensor for On-chip Potentiometric Determination of K+. Adv. Healthcare Mater. 2014, 3 (6), 876–881. 10.1002/adhm.201300541. 24376147

[ref70] Donnelly R. F. How Can Microneedles Overcome Challenges Facing Transdermal Drug Delivery?. Therapeutic Delivery 2017, 8 (9), 725–728. 10.4155/tde-2017-0028. 28825392

[ref71] Xie L.; Zeng H.; Sun J.; Qian W. Engineering Microneedles for Therapy and Diagnosis: A Survey. Micromachines 2020, 11 (3), 271 10.3390/mi11030271. 32150866 PMC7143426

[ref72] Qiao Y.; Li X.; Hirtz T.; Deng G.; Wei Y.; Li M.; Ji S.; Wu Q.; Jian J.; Wu F. Graphene-Based Wearable Sensors. Nanoscale 2019, 11 (41), 18923–18945. 10.1039/C9NR05532K. 31532436

[ref73] Azizi Machekposhti S.; Khanna S.; Shukla S.; Narayan R. Microneedle Fabrication Methods and Applications. MRS Commun. 2023, 13 (2), 212–224. 10.1557/s43579-023-00355-0.

[ref74] Sirbubalo M.; Tucak A.; Muhamedagic K.; Hindija L.; Rahić O.; Hadžiabdić J.; Cekic A.; Begic-Hajdarevic D.; Cohodar Husic M.; Dervišević A. 3D Printing—A “Touch-Button” Approach to Manufacture Microneedles for Transdermal Drug Delivery. Pharmaceutics 2021, 13 (7), 924 10.3390/pharmaceutics13070924. 34206285 PMC8308681

[ref75] Kjar A.; Huang Y. Application of Micro-Scale 3D Printing in Pharmaceutics. Pharmaceutics 2019, 11 (8), 390 10.3390/pharmaceutics11080390. 31382565 PMC6723578

[ref76] Prausnitz M. R. Engineering Microneedle Patches for Vaccination and Drug Delivery to Skin. Annu. Rev. Chem. Biomol. Eng. 2017, 8 (1), 177–200. 10.1146/annurev-chembioeng-060816-101514. 28375775

[ref77] Li Y.; Hu X.; Dong Z.; Chen Y.; Zhao W.; Wang Y.; Zhang L.; Chen M.; Wu C.; Wang Q. Dissolving Microneedle Arrays with Optimized Needle Geometry for Transcutaneous Immunization. European Journal of Pharmaceutical Sciences 2020, 151, 105361 10.1016/j.ejps.2020.105361. 32422374

[ref78] Loizidou E. Z.; Inoue N. T.; Ashton-Barnett J.; Barrow D. A.; Allender C. J. Evaluation of Geometrical Effects of Microneedles on Skin Penetration by CT Scan and Finite Element Analysis. Eur. J. Pharm. Biopharm. 2016, 107, 1–6. 10.1016/j.ejpb.2016.06.023. 27373753

[ref79] Makvandi P.; Kirkby M.; Hutton A. R. J.; Shabani M.; Yiu C. K. Y.; Baghbantaraghdari Z.; Jamaledin R.; Carlotti M.; Mazzolai B.; Mattoli V.; Donnelly R. F. Engineering Microneedle Patches for Improved Penetration: Analysis, Skin Models and Factors Affecting Needle Insertion. Nano-Micro Lett. 2021, 13 (1), 93 10.1007/s40820-021-00611-9. PMC8006208 34138349

[ref80] Romani N.; Thurnher M.; Idoyaga J.; Steinman R. M.; Flacher V. Targeting of Antigens to Skin Dendritic Cells: Possibilities to Enhance Vaccine Efficacy. Immunology and Cell Biology 2010, 88 (4), 424–430. 10.1038/icb.2010.39. 20368713 PMC2907485

[ref81] Martanto W.; Moore J. S.; Couse T.; Prausnitz M. R. Mechanism of Fluid Infusion during Microneedle Insertion and Retraction. J. Controlled Release 2006, 112 (3), 357–361. 10.1016/j.jconrel.2006.02.017. 16626836

[ref82] Zhu D. D.; Zhang X. P.; Zhang B. L.; Hao Y. Y.; Guo X. D. Safety Assessment of Microneedle Technology for Transdermal Drug Delivery: A Review. Advanced Therapeutics 2020, 3 (8), 2000033 10.1002/adtp.202000033.

[ref83] Kulkarni D.; Gadade D.; Chapaitkar N.; Shelke S.; Pekamwar S.; Aher R.; Ahire A.; Avhale M.; Badgule R.; Bansode R.; Bobade B. Polymeric Microneedles: An Emerging Paradigm for Advanced Biomedical Applications. Scientia Pharmaceutica 2023, 91 (2), 27 10.3390/scipharm91020027.

[ref84] Choi I.-J.; Kang A.; Ahn M.-H.; Jun H.; Baek S.-K.; Park J.-H.; Na W.; Choi S.-O. Insertion-Responsive Microneedles for Rapid Intradermal Delivery of Canine Influenza Vaccine. Journal of controlled release 2018, 286, 460–466. 10.1016/j.jconrel.2018.08.017. 30102940

[ref85] Li W.; Terry R. N.; Tang J.; Feng M. R.; Schwendeman S. P.; Prausnitz M. R. Rapidly Separable Microneedle Patch for the Sustained Release of a Contraceptive. Nature Biomedical Engineering 2019, 3 (3), 220–229. 10.1038/s41551-018-0337-4. 30948808

[ref86] Avcil M.; Çelik A. Microneedles in Drug Delivery: Progress and Challenges. Micromachines 2021, 12 (11), 1321 10.3390/mi12111321. 34832733 PMC8623547

[ref87] Sammoura F.; Kang J.; Heo Y.-M.; Jung T.; Lin L. Polymeric Microneedle Fabrication Using a Microinjection Molding Technique. Microsyst Technol. 2007, 13 (5), 517–522. 10.1007/s00542-006-0204-1.

[ref88] Wang Q.; Liu Q.; Zhong G.; Xu T.; Zhang X. Wearable Vertical Graphene-Based Microneedle Biosensor for Real-Time Ketogenic Diet Management. Anal. Chem. 2024, 96 (21), 8713–8720. 10.1021/acs.analchem.4c00960. 38745346

[ref89] Holicky M.; Fenech-Salerno B.; Cass A. E. G.; Torrisi F. Fabrication of Graphene Field Effect Transistors on Complex Non-Planar Surfaces. Appl. Phys. Lett. 2024, 125 (11), 113301 10.1063/5.0226780.

[ref90] Faraji Rad Z.; Prewett P. D.; Davies G. J. Parametric Optimization of Two-Photon Direct Laser Writing Process for Manufacturing Polymeric Microneedles. Additive Manufacturing 2022, 56, 102953 10.1016/j.addma.2022.102953.

[ref91] Gilpin V.; Surandhiran D.; Scott C.; Devine A.; Cundell J. H.; Gill C. I. R.; Pourshahidi L. K.; Davis J. Lasered Graphene Microheaters Modified with Phase-Change Composites: New Approach to Smart Patch Drug Delivery. Micromachines 2022, 13 (7), 1132 10.3390/mi13071132. 35888949 PMC9319399

[ref92] Cinti S.; Scognamiglio V.; Moscone D.; Arduini F. Efforts, Challenges, and Future Perspectives of Graphene-Based (Bio) Sensors for Biomedical Applications. Graphene bioelectronics 2018, 133–150. 10.1016/B978-0-12-813349-1.00006-8.

[ref93] Ren L.; Xu S.; Gao J.; Lin Z.; Chen Z.; Liu B.; Liang L.; Jiang L. Fabrication of Flexible Microneedle Array Electrodes for Wearable Bio-Signal Recording. Sensors 2018, 18 (4), 1191 10.3390/s18041191. 29652835 PMC5948552

[ref94] Novoselov K. S. Nobel Lecture: Graphene: Materials in the Flatland. Rev. Mod. Phys. 2011, 83 (3), 837–849. 10.1103/RevModPhys.83.837. 21732505

[ref95] Tian Z.; Cheng J.; Liu J.; Zhu Y. Dissolving Graphene/Poly (Acrylic Acid) Microneedles for Potential Transdermal Drug Delivery and Photothermal Therapy. J. Nanosci. Nanotechnol. 2019, 19 (5), 2453–2459. 10.1166/jnn.2019.15884. 30501739

[ref96] Patra S.; Choudhary R.; Madhuri R.; Sharma P. K. Graphene-Based Portable, Flexible, and Wearable Sensing Platforms: An Emerging Trend for Health Care and Biomedical Surveillance. Graphene Bioelectronics 2018, 307–338. 10.1016/B978-0-12-813349-1.00013-5.

[ref97] Kim T.; Cho M.; Yu K. J. Flexible and Stretchable Bio-Integrated Electronics Based on Carbon Nanotube and Graphene. Materials 2018, 11 (7), 1163 10.3390/ma11071163. 29986539 PMC6073353

[ref98] Lee H.; Choi T. K.; Lee Y. B.; Cho H. R.; Ghaffari R.; Wang L.; Choi H. J.; Chung T. D.; Lu N.; Hyeon T. A Graphene-Based Electrochemical Device with Thermoresponsive Microneedles for Diabetes Monitoring and Therapy. Nature Nanotechnol. 2016, 11 (6), 566–572. 10.1038/nnano.2016.38. 26999482

[ref99] Wang Z.; Xue L.; Li M.; Li C.; Li P.; Li H. Au@ SnO2-Vertical Graphene-Based Microneedle Sensor for in-Situ Determination of Abscisic Acid in Plants. Materials Science and Engineering: C 2021, 127, 112237 10.1016/j.msec.2021.112237. 34225877

[ref100] Keyvani F.; Zheng H.; Kaysir Md. R.; Mantaila D. F.; Ghavami Nejad P.; Rahman F. A.; Quadrilatero J.; Ban D.; Poudineh M. A Hydrogel Microneedle Assay Combined with Nucleic Acid Probes for On-Site Detection of Small Molecules and Proteins. Angew. Chem., Int. Ed. 2023, 62 (21), e202301624 10.1002/anie.202301624. 36946837

[ref101] Gao A.; Chen Y.; Liang H.; Cui X.; Zhang A.; Cui D. Developing an Efficient MGCR Microneedle Nanovaccine Patch for Eliciting Th 1 Cellular Response against the SARS-CoV-2 Infection. Theranostics 2023, 13 (14), 4821–4835. 10.7150/thno.83390. 37771766 PMC10526668

[ref102] Jin Q.; Chen H.-J.; Li X.; Huang X.; Wu Q.; He G.; Hang T.; Yang C.; Jiang Z.; Li E. Reduced Graphene Oxide Nanohybrid–Assembled Microneedles as Mini-invasive Electrodes for Real-time Transdermal Biosensing. Small 2019, 15 (6), 1804298 10.1002/smll.201804298. 30605244

[ref103] Gaware S. A.; Rokade K. A.; Bala P.; Kale S. N. Microneedles of Chitosan-porous Carbon Nanocomposites: Stimuli (pH and Electric Field)-initiated Drug Delivery and Toxicological Studies. J. Biomed. Mater. Res., Part A 2019, 107 (8), 1582–1596. 10.1002/jbm.a.36672. 30884173

[ref104] Zhang W.; Liu N.; Zhang Q.; Jiang W.; Wang Z.; Zhang D. Stimuli-Responsive Polymer Microneedle System for Transdermal Drug Delivery. Progress in Chemistry 2023, 35 (5), 735 10.7536/PC220710.

[ref105] Jiang X.; Xia W.; Pan J.; Yang W.; Zhang S.; Li C.; Zan T.; Lai Y.; Xu Z.; Yu H. Engineered Microneedle Systems for Topical Cancer Therapy. Applied Materials Today 2023, 31, 101774 10.1016/j.apmt.2023.101774.

[ref106] Liu X.; Song H.; Sun T.; Wang H. Responsive Microneedles as a New Platform for Precision Immunotherapy. Pharmaceutics 2023, 15 (5), 1407 10.3390/pharmaceutics15051407. 37242649 PMC10220742

[ref107] Xie R.; Li W.; Shi K.; Yang L.; Chen H.; Jiang S.; Zeng X. Versatile Platforms of Microneedle Patches Loaded with Responsive Nanoparticles: Synthesis and Promising Biomedical Applications. Advanced NanoBiomed Research 2024, 4 (4), 2300142 10.1002/anbr.202300142.

[ref108] Jung S.; Chang S.; Kim N.-E.; Choi S.-O.; Song Y.-J.; Yuan Y.; Kim J. Curcumin/Zeolitic Imidazolate Framework-8 Nanoparticle-Integrated Microneedles for pH-Responsive Treatment of Skin Disorders. ACS Appl. Nano Mater. 2022, 5 (9), 13671–13679. 10.1021/acsanm.2c03884.

[ref109] Lan X.; She J.; Lin D.; Xu Y.; Li X.; Yang W.; Lui V. W. Y.; Jin L.; Xie X.; Su Y.-X. Microneedle-Mediated Delivery of Lipid-Coated Cisplatin Nanoparticles for Efficient and Safe Cancer Therapy. ACS Appl. Mater. Interfaces 2018, 10 (39), 33060–33069. 10.1021/acsami.8b12926. 30204401

[ref110] Makvandi P.; Jamaledin R.; Chen G.; Baghbantaraghdari Z.; Zare E. N.; Di Natale C.; Onesto V.; Vecchione R.; Lee J.; Tay F. R. Stimuli-Responsive Transdermal Microneedle Patches. Mater. Today 2021, 47, 206–222. 10.1016/j.mattod.2021.03.012. PMC9635273 36338772

[ref111] Fan Z.; Wei Y.; Yin Z.; Huang H.; Liao X.; Sun L.; Liu B.; Liu F. Near-Infrared Light-Triggered Unfolding Microneedle Patch for Minimally Invasive Treatment of Myocardial Ischemia. ACS Appl. Mater. Interfaces 2021, 13 (34), 40278–40289. 10.1021/acsami.1c09658. 34424666

