# Correction to “Graphene-Based Polymeric Microneedles for Biomedical Applications: A Comprehensive Review”

**DOI:** 10.1021/acsabm.5c02468

**Published:** 2026-01-02

**Authors:** Somayeh Moradi, Faezeh Nargesi Azam, Hossein Abdollahi, Nariman Rajabifar, Amir Rostami, Pablo Guzman, Payam Zarrintaj, Seyed Mohammad Davachi

**Affiliations:** † Department of Polymer Engineering, Faculty of Engineering, 117045 Urmia University, Urmia 57561-51818, Iran; ‡ Polymer Engineering Department, Faculty of Chemical Engineering, 41616 Tarbiat Modares University, Tehran 14115-114, Iran; § Department of Polymer Engineering and Color Technology, 48410 Amirkabir University of Technology, Tehran 15875-4413, Iran; ∥ Department of Chemical Engineering, Faculty of Petroleum, Gas and Petrochemical Engineering, 199786 Persian Gulf University, Bushehr 75169-13817, Iran; ⊥ Department of Biology and Chemistry, 4420 Texas A&M International University, Laredo, Texas 78041, United States; # Independent Researcher, E3 LLC, Louisville, Kentucky 40059, United States

This Addition and Correction addresses an author affiliation correction to the originally published article.

**Original affiliation for Payam Zarrintaj:**

Department of Biology and Chemistry, Texas A&M International University, Laredo, Texas 78041, United States

**Corrected affiliation for Payam Zarrintaj:**

Independent Researcher, E3 LLC, Louisville, Kentucky 40059, United States

The affiliations of all other authors are unchanged from those reported in the original publication.

This correction is limited to the author affiliation information and does not affect the scientific content, data interpretation, or conclusions of the article.

